# Autophagic degradation of PML promotes susceptibility to HSV-1 by stress-induced corticosterone

**DOI:** 10.7150/thno.46921

**Published:** 2020-07-11

**Authors:** Wen Li, Zhuo Luo, Chang-Yu Yan, Xiao-Hua Wang, Zheng-Jie He, Shu-Hua Ouyang, Chang Yan, Li-Fang Liu, Qing-Qing Zhou, Han-Lu Mu, Hai-Biao Gong, Wen-Jun Duan, Lei Liang, Hiroshi Kurihara, Du Feng, Yi-Fang Li, Rong-Rong He

**Affiliations:** 1 Guangdong Engineering Research Center of Chinese Medicine & Disease Susceptibility, Jinan University, Guangzhou 510632, China.; 2 International Cooperative Laboratory of Traditional Chinese Medicine Modernization and Innovative Drug Development of Chinese Ministry of Education (MOE), College of Pharmacy, Jinan University, Guangzhou 510632, China.; 3 Guangdong Province Key Laboratory of Pharmacodynamic Constituents of TCM and New Drugs Research, College of Pharmacy, Jinan University, Guangzhou 510632, China.; 4 Affiliated Cancer Hospital & Institute of Guangzhou Medical University, Guangzhou; Municipal and Guangdong Provincial Key Laboratory of Protein Modification and Degradation, School of Basic Medical Sciences, Guangzhou Medical University, 511436, Guangzhou, China.; 5 State Key Laboratory of Respiratory Disease, Guangzhou Medical University, 511436, Guangzhou, China.

**Keywords:** Stress, CORT, HSV-1, Autophagy, PML

## Abstract

**Rationale:** Herpes simplex virus type 1 (HSV-1) is a neurotropic virus that can cause a variety of clinical syndromes including mucocutaneous disease and HSV-1 encephalitis (HSE). Here, we characterize the molecular mechanisms underlying the susceptibility to HSV-1 under stressful conditions.

**Methods:** Restraint stress and corticosterone (CORT, a primary stress hormone) were respectively used to establish HSV-1 susceptible model *in vivo* and *in vitro*. Viral titers were determined by plaque assay. Western blotting, immunofluorescence, transmission electron microscopy (TEM), qRT-PCR, H&E staining, IHC staining and flow cytometry were employed to evaluate virus-related protein expressions and detect the activation of autophagy. Loss- and gain-function assays, co-immunoprecipitation (co-IP) technique and autophagy agonist/antagonist treatments were applied in mechanistic experiments.

**Results:** Restraint stress increased the susceptibility of mouse brain to HSV-1. Similarly, CORT treatment enhanced the susceptibility of neural cells to HSV-1. Furthermore, PML protein level in HSV-1 infected brain tissues and neural cells was remarkably decreased by stress treatment *in vivo* or CORT treatment *in vitro*, while its transcriptional level was not affected. Notably, a striking decline in protein expressions of ICP27 and gB was observed in PML-overexpressing cells, which was reversed by CORT treatment. By contrast, protein expression of gB was increased by knockdown with si-*PML* in virus-infected SH-SY5Y cells. We further discovered that CORT-driven PML degradation was dependent on the activation of autophagy in a ULK1-independent manner, rather than proteasome pathway. Bafilomycin A1 (BaF1) attenuated the augmentation effect of CORT on HSV-1 infection. The expressions of viral proteins were reduced in LC3-depleted cells, and the degradation of PML by CORT-induced autophagy was prevented in cells with LC3 knockdown by RNAi. Interestingly, PML was revealed to interact with the autophagic cargo receptor P62 and the autophagic effector protein LC3. Additionally, CORT failed to increase gB protein level when PML was silenced, providing direct evidence linking autophagic degradation of PML and CORT-induced virus susceptibility.

**Conclusion:** Our results revealed that restraint stress/CORT increased HSV-1 susceptibility by delivering PML into autolysosomes for degradation. The results obtained from *in vitro* and *in viv*o models not only demonstrated the adverse effects of stress on HSV-1 infection, but also systematically investigated the underlying molecular mechanisms. These discoveries broaden our understanding of the interplay between host and viruses, and a comprehensive understanding of the role of autophagy in viral infection will provide information for future development of innovative drugs against viral infection.

## Introduction

HSV-1 is a common human pathogen that causes cold sores and HSE 1. HSV-1 establishes a lifelong latency in sensory neurons of the trigeminal ganglia after primary infection in epithelial cells. Under stress conditions, the virus can exit the latent state and enter the replication cycle to spread 2-5. Clinical studies have also demonstrated that psychological stresses increase the recurrence of HSV-1 and HSV-2 6, 7. It has been revealed that stress increases corticosteroid levels, leading to activation of the glucocorticoid receptor (GR). The activated GR stimulates HSV-1 productive infection, in part because the ICP0 promoter is cooperatively transactivated by the GR and Krüppel-like transcription factor 15 (KLF15) 8.

The nervous system has a limited capability to regenerate, and therefore it relies on autophagy and intrinsic immunity to restrict replication and spread of HSV-1, thus protecting neuron cells from destructive damage induced by interferons (IFNs) 9, 10. Autophagy is a highly conserved cellular process that degrades cytoplasmic materials and damaged organelles through the autophagosome-lysosome system 11, 12. Studies have indicated that autophagy can exert both antiviral and proviral effects 13-16. Autophagy may be directly involved in the degradation of phagocytosed viral particles, or it may enhance the presentation of endogenous viral antigens on MHC class I molecules during HSV-1 infection 17. To cope with this scavenging effect of autophagy on virus, HSV-1 has evolved mechanisms to favor its own intracellular replication or its transmission to new hosts. Viral proteins such as ICP34.5 and Us11 have been reported to function as anti-autophagic factors by targeting Beclin-1 and protein kinase R (PKR), respectively 18-20. Hence, there is growing interest in autophagy and HSV-1 replication.

Intrinsic immunity refers to a form of innate immunity that immediately and directly restricts viral replication and assembly, thereby rendering a cell nonpermissive to a specific class or species of viruses 21. Promyelocytic leukemia protein-nuclear bodies (PML-NBs) contribute to intrinsic immunity against a variety of viruses 22-24. PML-NBs are dynamic cellular structures composed of PML, Daxx (death domain associated protein), the nuclear antigen SP100, and the chromatin remodeler ATRX (alpha-thalassemia mental retardation X-linked protein). PML, also known as TRIM19, plays important roles in genome stability, programmed cell death, and tumorigenesis 25-27. Interestingly, several lines of evidences indicate the important role of PML in combating HSV-1 infection 28, 29. During HSV-1 infection, viral protein ICP0 induces the degradation of PML 30, 31. For decades, the ubiquitin-proteasome pathway and SUMO modification have been implicated as the main mechanisms involved in PML degradation 32-34. However, the precise molecular mechanisms that coordinate PML and autophagy in HSV-1 susceptibility induced by stress remain to be determined.

In this study, we investigated the cross-talk between autophagy and PML with a focus on understanding its role in inducing HSV-1 susceptibility under stress. Interestingly, we found that autophagy was activated upon stress exposure, and this caused autophagy-dependent degradation of PML, which contributed to the susceptibility to HSV-1 under stress.

## Materials and Methods

### Cells and transfection

SH-SY5Y, TM4, HT22, BV-2 and Vero cells (African green monkey kidney) used in this research were kept in our laboratory. NRK cells, GFP-LC3-LAMP1-Cherry-NRK cells, ULK1^-/-^ MEFs, ULK1 Ctrl MEFs, WT HeLa cells and Beclin-1 KD HeLa cells used in this paper were obtained from Prof. Du Feng (School of Basic Medical Sciences, Guangzhou Medical University). All these cells were cultured in DMEM (Gibco, Grand Island, NY, USA) supplemented with 10% fetal bovine serum (ThermoFisher Scientific, Franklin, MA, USA) at 37 °C with 5% CO_2_. For transfection, Lipofectamine 2000 (Invitrogen, 11668027) was used with reference to the manufacturer's instructions.

### Reagents and antibodies

CORT (Sigma, 27840), RU-486 (Mifepristone, Sigma, M8046), 3-MA (M9281), cycloheximide (CHX, C4859) and BaF1 (B1793) were purchased from Sigma. Rapamycin (S1039) and MG132 (S2619) were purchased from Selleck. The following antibodies: anti-HSV-1 + HSV-2 gB (10B7) (Abcam, ab6506), anti-HSV-1+HSV-2 ICP27 antibody(1-L-11) (Abcam, ab31631), anti-HSV-1 ICP8 major DNA binding protein antibody (11E2) (Abcam, ab20194), anti-ICP0 antibody (Abcam, ab6513), anti-PML (Abcam, ab96051), anti-PML (Proteintech, 21041-1-AP), anti-ULK1 (Sigma, A7481), anti-ATG5 (C-terminal) (Sigma, A0731), anti-LC3B antibody (Sigma, L7543), anti-LC3B antibody (CST, 2775), anti-P62 (Abcam, ab56416), anti-Beclin-1 (Santa Cruz, sc-11427), anti-HA (Abcam, ab9110), anti-TMEM173 (anti-STING, Proteintech, 19851-1-AP), anti-MAVS (Proteintech, 14341-1-AP), anti-IFNβ (Abcam, ab85803), anti-cGAS (Abcam, ab176177), anti-phospho-TBK1 (CST, 5483), anti-TBK1 (CST, 3504), anti-GAPDH (Transgen, HC301), anti-β-Actin mouse monoclonal antibody (Transgen, HC201), goat anti-mouse IgG-HRP (FUDE, FDM007) and goat anti-rabbit IgG-HRP (FUDE, FDR007) were used for immunoblot analysis.

### Plasmids and siRNAs

GFP-LC3 and Flag-ULK1 were maintained in our laboratory. Cherry-PML [NM_033238.2] */HA and PML [NM_033238.2] */HA were produced by VectorBuilder. Details of the vectors can be found at https://en.vectorbuilder.com/vector/VB180412-1110wye.html and https://en.vectorbuilder.com/vector/VB191224-2235bfu.html. SiRNA sequences corresponding to LC3 (si-*LC3*) and PML (si-*PML*) were synthesized by Guangzhou RiboBio Co., Ltd. The target sequences were as follows: GTCTACGCCTCCCAAGAAA (si-*LC3*, #1), CACCCATCGCTGACATCTA (si-*LC3*, #2), CCTTCTTCCTGCTGGTCAA (si-*LC3*, #3). GAGCTCAAGTGCGACATCA (si-*PML*, #1), GACCTCAGCTCTTGCATCA (si-*PML*, #2), CGCCCTGGATAACGTCTTT (si-*PML*, #3). The negative control was NC, provided by Guangzhou Ruibo.

### Primary culture of mouse cortical neurons

Primary mouse cortical neurons were prepared as reported previously 35. In brief, neonatal mice were euthanized by decapitation and intact hemispheres were separated from the head. After removing meninges and hippocampus using a stereoscope, the cortex was dissected out and trypsinized in 0.08% trypsin with DNase I (Sigma-Aldrich, DN25) for 20 min at 37 °C. Dissociated neuronal cells were cultured in serum-free Neurobasal^TM^ medium (Gibco, 21103-049) with supplement of 2% B27 (Gibco, 17504-044), 1% Glutamax (Gibco, 35050-061) and penicillin and streptomycin using the dishes coated with poly-L-lysine. And then half of the medium was replaced with fresh medium twice a week. Primary neurons were used for experiments 11 day after being cultured.

### Animals and HSV-1 infection model

**Figure 1:**
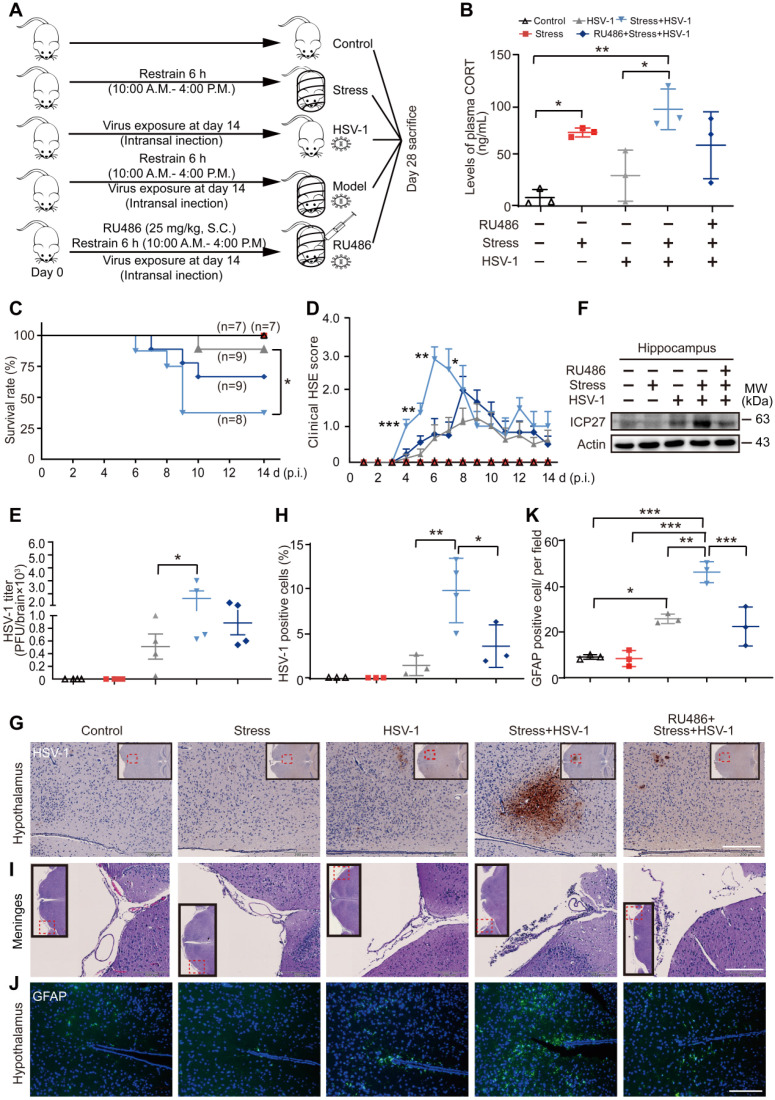
** Restraint stress increases the susceptibility of mouse brain to HSV-1.** (***A***) The experimental procedures for determining restraint stress-induced susceptibility to HSV-1 in mice. Mice were intranasally infected with 10^6^ PFU of HSV-1 in 20 µL infection medium (DMEM) at day 14. Details of the mouse model are described in the Method section. HSV-1 strain F viral titer: 5×10^7^ PFU/mL. (***B***) Plasma CORT level was detected by liquid chromatographic assay using cortisol as the internal standard. Data were analyzed by one-way ANOVA and Tukey's multiple comparisons test was used to assess the statistical significance. Data were presented as mean ± SD. n=3 mice. ^*^P < 0.05, ^**^P < 0.01 *vs.* indicated group. (***C***) Statistical analysis of survival rate. Survival of the mice was recorded up to 14 days p.i. Survival rate was analyzed with the Kaplan-Meier method and Log-rank (Mantel-Cox) test. Data were presented as mean ± SD. n=7~9 mice. ^*^P < 0.05 *vs.* indicated group. (***D***) Clinical HSE score. Mice were examined daily for symptoms of HSE after administration of HSV-1. Data were analyzed by one-way ANOVA and Tukey's multiple comparisons test was used to assess the statistical significance. Data were presented as mean ± SD. n=7~9 mice. ^*^P < 0.05, ^**^P < 0.01, ^***^P < 0.001 *vs.* the HSV-1 group. (***E***) Viral titers in mouse brain were quantified by the plaque assay. Data were analyzed by one-way ANOVA and Tukey's multiple comparisons test was used to assess the statistical significance. Data were presented as mean ± SD. n=4 mice. ^*^P < 0.05 *vs.* indicated group. (***F***) Proteins were extracted from mouse hippocampus for immunoblot analysis of ICP27. Actin was used as the loading control. (***G***) Representative IHC staining of mouse brain paraffin sections (brown, HSV-1). The insets showed low-magnification views, and the red dotted boxes indicated the areas that were enlarged in the main images. Scale bar, 200 µm. n=3~4 mice. (***H***) HSV-1 positive cells in hypothalamus were analyzed by one-way ANOVA and Tukey's multiple comparisons test was used to assess the statistical significance. Data were presented as mean ± SD. n=3~4 mice.^ *^P < 0.05, ^**^P < 0.01* vs.* indicated group. (***I***) Representative H&E staining to assay pathological changes in the meninges. The red dotted boxes in the insets indicated the areas that were enlarged in the main images. Scale bar, 200 µm. n=3~4 mice. (***J***) Representative immunofluorescence staining of mouse brain paraffin sections (green, GFAP; blue, DAPI). Scale bar, 200 µm. n=3 mice. (***K***) GFAP positive cells in hypothalamus were analyzed by one-way ANOVA and Tukey's multiple comparisons test was used to assess the statistical significance. Data were presented as mean ± SD. n=3 mice. ^*^P < 0.05, ^**^P < 0.01, ^***^P < 0.001* vs.* indicated group.

BALB/C mice (4-week old, male) were divided into five groups: control group, restraint stress group, HSV-1 group, model group (restraint stress + HSV-1) and RU486 group (RU486 + restraint stress + HSV-1). The experimental approach for restraint stress or/and HSV-1 infection (HSV-1 strain F) is shown in Figure 1A. To apply stress, mice were restrained daily for 6 h (from 10:00 a.m. to 4:00 p.m.) until the 14th day. On the 14th day, mice were intranasally infected with 10^6^ PFU of HSV-1 in 20 μL infection medium (DMEM). After HSV-1 infection, mice were kept under restraint conditions for 6 h (from 10:00 a.m. to 4:00 p.m.) daily until they were sacrificed. In the RU486 group, 25 mg/kg RU486 was subcutaneously injected 2 h before restraint, daily. All mice were sacrificed at day 28. Mice were scored for disease and weighed at the indicated time. The clinical HSE scoring was performed in a blinded manner, according to previous studies by others 36, 37. The experimental procedures on animals were conducted in accordance with the Guide for the Care and Use of Laboratory Animals in Jinan University as adopted and promulgated by the United States National Institutes of Health. The HSV-1-related pathogenic operations were performed in the Animal Biosafety Level 2 Laboratory.

### Plasma CORT determination

Blood samples were transferred into heparinized tubes and centrifuged at 2400 g for 10 min. The separated plasma was collected and stored at -80 °C for further analysis. Plasma CORT content was determined by HPLC-UV. Briefly, plasma was mixed with cortisone as an internal standard. Steroids were extracted by adding acetic ether and mixed by vortexing. The mixture was immediately centrifuged at 800 g for 5 min. The organic phase was washed with water, centrifuged and evaporated under nitrogen. The residue was redissolved in the mobile phase (acetonitrile/water: 38/62, v/v). CORT determination was performed in a HPLC system with a UV detector at 254 nm at a flow rate of 1 mL/min. CORT and cortisone standards were purchased from Sigma (St. Louis, USA).

### Viruses and virus plaque assays

HSV-1 strain F and HSV-1-EGFP were amplified in Vero cells with DMEM (Gibco) containing 2% FBS. At 48 h post-infection (p.i.), supernatants from infected Vero cells were collected when the cytopathic effect (CPE) was more than 80%. The supernatants were frozen and thawed twice, and then centrifuged at 1300 g for 15 min at 4 °C to remove the precipitate in the supernatant. An ultracentrifugation of the supernatant at 10,000 g for 1 h at 4 °C was required to obtain virus particles.

Viral titers in the supernatants and cells were determined by standard plaque assay. Briefly, monolayers of Vero cells were infected with serially diluted HSV-1 (in DMEM) for 2 h. Subsequently, the viral suspensions were removed. DMEM containing 1% methylcellulose with 2% FBS was added, allowing the virus to spread only via the cell to-cell route. After 48~72 h of infection, the number of plaques in each well was counted by crystal violet staining. Three parallel assays were performed in each group. HSV-1 strain F viral titer: 5×10^7^ PFU/mL. HSV-1-EGFP viral titer: 1×10^8^ PFU/mL.

### Quantification of virus in mouse brain by plaque assay

Mouse brains were collected and frozen in tubes with 1 mL medium/tube. The tissues were homogenized, thawed, and frozen again. Tissue homogenates were centrifuged at 1300 g for 5 min at 4 °C. The resulting supernatants were collected and added to one well of Vero cell monolayers in 24-well plates seeded the day before. After 2 h incubation, Vero cell monolayers were washed once with PBS, overlaid with medium containing 1% methylcellulose plus with 2% FBS for 4 days, and stained to count plaques.

### Detection of HSV-1-EGFP by cytometer

Primary mouse cortical neurons were treated with 10 μM CORT or not for 48h, or subjected to 10 μM CORT in the presence of RU486 for 48 h, then subjected to HSV-1 infection at a multiplicity of infection (MOI) of 1. At 24 h p.i., cells were trypsinized, washed and resuspended in sheath fluid. Flow cytometry was performed using the EPICS XL flow cytometer (Beckman Coulter) in FL1 channel (Ex 488 nm, Em 525 nm) for EGFP signal detection.

### Immunoblot analysis

Treated or infected cells were washed once with PBS. Whole-cell lysates used for immunoblot analysis were prepared in lysis buffer (Beyotime, P0013) containing a protease inhibitor cocktail tablet (Roche, 4693116001) on ice. After quantification of total cellular protein using a protein assay kit (Pierce, 23225), whole-cell lysates were collected in SDS-PAGE loading buffer (FUDE, FD002). Proteins were separated by gel electrophoresis, followed by an electrophoretic transfer onto PVDF membranes. Membranes were blocked in TBST with 5% non-fat milk for one hour at room temperature. Corresponding primary antibodies were used for overnight incubation at 4 °C. The membranes were washed to remove the primary antibodies, then incubated with HRP-labeled secondary antibodies at room temperature for 2 h. Immunoreactivity was detected with an immobilon^TM^ western chemiluminescent HRP substrate kit (Millipore, WBKLS0500). Membranes were imaged by an automatic chemiluminescence image analysis system (Tanon 5200). GAPDH or β-actin (Actin) was used as a quantitative reference. Band intensity was quantified by ImageJ software.

### Immunofluorescence staining of cells and confocal microscopy

Cells were seeded on a coverslip to 60% confluence and then transfections were conducted with reference to the suppliers's instruction manual. GFP-LC3 (1.5 μg) and Cherry-PML (1.5 μg) were transfected to each well of a 6-well plate. For immunofluorescence staining, cells were fixed with freshly prepared 4% formaldehyde at 37 °C for 15 min. After washing with PBS, DAPI (Beyotime, C1002) staining was performed to distinguish the morphology of the nucleus from the cytoplasm. Antifade mounting medium was applied then coverslips were mounted with nail enamel. Coverslips were preserved at 4 °C in the dark. Cell images were captured with a Zeiss800 confocal microscope.

Cells were grown to 60% confluence on a coverslip. After the indicated treatment, cells were washed twice with PBS and fixed with freshly prepared 4% formaldehyde at 37 °C for 15 min. Antigen accessibility was increased by treatment with 0.2% Triton X-100. Cells were incubated with anti-LC3B antibody (CST, 2775, 1:100) and anti-HSV-1 + HSV-2 gB (10B7) (Abcam, ab6506, 1:200) or anti-PML (Proteintech, 21041-1-AP, 1: 100) at 4 °C overnight. After washing with PBS, cells were stained with Alexa Fluor 555-labeled donkey anti-mouse IgG antibodies (Invitrogen, A31570, 1:400) and Alexa Fluor 488-labeled donkey anti-rabbit IgG antibodies (Invitrogen, A21206, 1:300) or Alexa Fluor 594-labeled donkey anti-rabbit IgG antibodies (Invitrogen, A32754, 1:300) for 1 h at room temperature. Nuclei were stained with DAPI for 10 min. The sections were washed five times in PBS and mounted using antifade medium. Images were captured with a Zeiss800 confocal microscope.

### qRT-PCR gene expression analysis

Cells were subjected to the indicated treatments. Gene expression levels were measured by qRT-PCR using gene-specific primers as in Table 1. In brief, total RNA was isolated with TRIzol (Life Technologies), followed by a DNase I (Beyotime, D7076) treatment to eliminate contaminating genomic DNA. Reverse transcription reactions were performed using a RevertAid First Strand cDNA Synthesis Kit (Thermo, K1622) according to the manufacturer's protocol. Two-step quantitative RT-PCR was applied to determine relative gene expression using TransStart Top Green qPCR SuperMix (Transgen, AQ131). All genes tested were amplified on a CFX Connect™ Real-Time system (Bio-Rad, Hercules, CA, USA). The expression of related genes was normalized to the house keeping gene 18S. Relative mRNA expression levels were calculated by the 2^-△△Ct^ method.

### Transmission electron microscopy

Cells were cultured and treated as described above. For electron microscopy, cells were collected by centrifugation at 800 g for 3 min. The cell pellet was gently washed once with PBS then centrifuged again at 800 g for 3 min to get the cell pellet. Cells were fixed overnight at 4 °C with 2.5% glutaraldehyde (Millipore, 1.04239.1000-1L) in PBS, and then dehydrated in a graded ethanol series and embedded. Approximately 70 nm ultrathin sections were mounted on nickel grids. The samples were then stained and visualized using a 120 kV Jeol electron microscope (JEM-1400) at 80 kV. Images were captured using a Gatan-832 digital camera.

### H&E staining

Brain tissues were fixed in phosphate-buffered 4% paraformaldehyde, embedded in paraffin and sliced into 5 μm sections. H&E staining was performed by a commercial kit (Beyotime Biotechnology, Shanghai, China). Briefly, after deparaffinization and rehydration, slides were stained by hematoxylin and eosin according to the manufacturer's instructions. Morphological evaluation of samples was conducted with an automatic scanning microscope (PreciPoint, Freising, Germany).

### IHC staining

Paraffin-embedded sections at a thickness of 5 μm were dewaxed in xylene and rehydrated in an alcohol gradient. Antigen retrieval was conducted with 0.01 M sodium citrate buffer (pH 6.0) by boiling the slices in a microwave for 20 min. Sections were placed in 3% hydrogen peroxide for 30 min to remove endogenous peroxidase activity. To prevent non-specific binding, the sections were blocked by 10% goat serum for 1 hour at room temperature in a humidified chamber. Then, primary antibodies (anti-HSV, Abcam, ab9533, 1: 100; anti-PML, Proteintech, 21041-1-AP, 1: 100) were added to sections for an incubation at 4 °C overnight. After several washes in PBS, slides were covered with the corresponding secondary antibody at room temperature for 60 min. The secondary antibody was washed off then DAB staining was performed for 10 min in the dark. To visualize nuclei, hematoxylin was added for 5 min. After being dehydrated in an alcohol gradient and vitrified by dimethylbenzene, slides were mounted with coverslips using neutral gum and detected by an automatic scanning microscope (PreciPoint, Freising, Germany).

### Immunofluorescence staining of tissue sections

Paraffin-embedded sections (5 μm thick) were subjected to dewaxing, rehydration, antigen retrieval, hydrogen peroxide treatment, and blocking of non-specific binding sites as described above. Anti-GFAP antibody (Proteintech, 60190-1-Ig, 1: 100) was then added to sections for an incubation at 4 °C overnight. After several washes in PBS, slides were incubated with Alexa Fluor 488-labeled donkey anti-mouse IgG antibody (Invitrogen, A-21202, 1: 300) for 1 h at room temperature. Nuclei were stained with DAPI for 10 min. The sections were washed five times in PBS and mounted using antifade medium. Sections were imaged on a Zeiss800 confocal microscope.

### Co-IP

SH-SY5Y cells were seeded into a 10 cm dish, and treated with or without CORT (50 μM for 48 h). Cells were lysed with cell lysis buffer for western blotting and IP (Beyotime Biotechnology, China) with PMSF and protease inhibitor. Briefly, anti-LC3 or anti-P62 antibody was added for 12 h into cell lysates, and agarose-protein A/G beads were added subsequently for another 12 h. Beads were washed five times with cell lysis buffer. Finally, LC3- or P62- coprecipitated proteins were subjected to immunoblot analysis with anti-PML antibody.

### Statistical analysis

All analyses were performed using GraphPad Prism 7 software (GraphPad Software Inc, San Diego, CA, USA). One-way ANOVA followed by Dunnett's multiple comparisons test, one-way ANOVA followed by Tukey's multiple comparisons test and a non-parametric Wilcoxon Mann-Whiney U-test were used for statistical analyses. All data are expressed as means ± SD. *P*-values of less than 0.05 are considered statistically significant.

## Results

### Restraint stress increases the susceptibility of mouse brain to HSV-1

We have previously demonstrated that psychological stress simulated by restraint could be used to increase the susceptibility to H1N1 38, 39, but it was still unclear if stress could also promote HSV-1 susceptibility. To this end, mice were challenged with restrained stress and/or HSV-1 according to the experimental procedures (Figure 1A). Consistently with our previous study 40, the plasma level of CORT, a stress-induced hormone, was increased significantly after restraint stress loading (Figure 1B). A significantly lowered survival rate was observed in the model group (Stress + HSV-1) as compared with the HSV-1 group (Figure 1C). Moreover, the average HSE score of mice in all HSV-1 infected groups began to increase on day 4, and the model group showed significantly higher HSE scores than the virus alone group from day 4 to day 7 (Figure 1D). Plaque assay revealed that the HSV-1 titer in the whole brain tissue of the model group was significantly higher than that of the virus alone group (Figure 1E). ICP27 is a multifunctional viral protein primarily involved in viral replication, late gene expression, and reactivation from latency. ICP27 protein expression in hippocampus of the model group increased markedly compared with the virus only group (Figure 1F).

Moreover, immunohistochemistry (IHC) staining of HSV-1 antigen showed significantly increased replication of HSV-1 in the model group (Figure 1G-H). Hematoxylin-eosin (H&E) staining of brain paraffin sections revealed that the model group had severer inflammatory infiltration of monocytic cells in the meninges than the virus only group (Figure 1I). Additionally, immunofluorescence (IF) staining was used to detect inflammatory infiltration at the molecular level using an antibody raised specifically against glial fibrillary acidic protein (GFAP, green). We detected a moderate number of positively stained cells in brain tissue around the sagittal suture in the virus group, and a high level of positive cells in the model group (Figure 1J-K). RU486 (mifepristone) is a derivative of the progestin norethindrone, and acts as a glucocorticoid receptor (GR) antagonist 41. Interestingly, the plasma CORT content, survival rate, clinical HSE score, HSV-1 titer in the brain, protein expression of ICP27, inflammatory infiltration of monocytic cells in the meninges, HSV-1 antigen expression in the hypothalamus and GFAP positively-stained cells in brain tissues of the model group were all alleviated by RU486 (Figure 1B-K). Collectively, these results suggest that restraint stress increases the susceptibility of mice to HSV-1 by promoting CORT production.

### CORT enhances the susceptibility of cells to HSV-1

**Figure 2:**
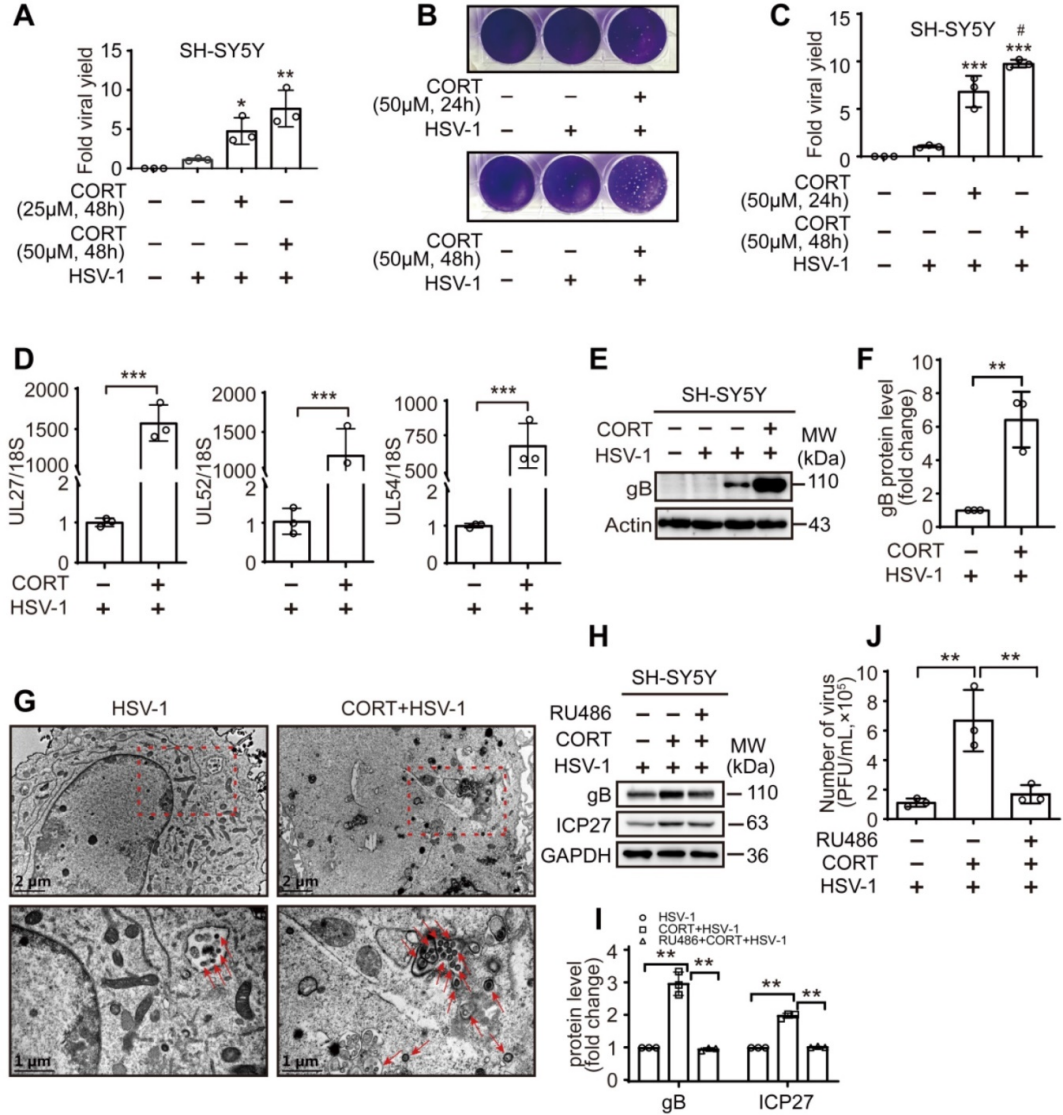
** CORT enhances the susceptibility of cells to HSV-1.** (***A***) SH-SY5Y cells were subjected to CORT according to the indicated concentrations for 48 h, and then were infected with HSV-1 (MOI=1) and harvested at 24 h p.i. HSV-1 strain F viral titer: 5×10^7^ PFU/mL. Viral productions were quantified by plaque assay. Data were analyzed by one-way ANOVA and Dunnett's multiple comparisons test was used to assess the statistical significance. Data were presented as mean ± SD of three independent experiments. ^*^P < 0.05, ^**^P < 0.01* vs.* the HSV-1 group. (***B*, *C***) SH-SY5Y cells were subjected to 50 µM CORT for 24 h or 48 h, and then were infected with HSV-1 (MOI=1) and harvested at 24 h p.i. HSV-1 strain F viral titer: 5×10^7^ PFU/mL. Viral productions were quantified by plaque assay. Data were analyzed by one-way ANOVA and Tukey's multiple comparisons test was used to assess the statistical significance. Data were presented as mean ± SD of three independent experiments. ^***^P < 0.001 *vs*. the HSV-1 group. ^#^*P* < 0.05 *vs*. the CORT (24 h) + HSV-1 group. (***D***) SH-SY5Y cells were treated with 50 µM CORT or not for 48h, then infected with HSV-1 for 24 h (MOI=1). HSV-1 strain F viral titer: 5×10^7^ PFU/mL. The expression level of corresponding viral genes was determined by real-time PCR. A non-parametric Wilcoxon Mann-Whiney U-test was used for statistical analyses. Data were presented as mean ± SD of three biological samples. ^***^P < 0.001 *vs.* indicated group. (***E***) Immunoblot analysis of gB and Actin. SH-SY5Y cells were incubated with 50 µM CORT or not for 48 h, and then were infected with HSV-1 (MOI=1) and harvested at 24 h p.i. HSV-1 strain F viral titer: 5×10^7^ PFU/mL. Immunoblot was performed in three independent experiments, and representative blotting results were shown. (***F***) The fold change of gB protein level was analyzed by a non-parametric Wilcoxon Mann-Whiney U-test. Data were presented as mean ± SD of three independent experiments. ^**^P < 0.01 *vs.* indicated group. (***G***) SH-SY5Y cells were incubated with 50 µM CORT or not for 48 h, and then were infected with HSV-1 (MOI=1) for 24 h. HSV-1 strain F viral titer: 5×10^7^ PFU/mL. Samples were analyzed by TEM. Red dashed frames demarcated the enlarged areas. Red arrows indicated HSV-1 viral particles. Scale bars, 1 µm, 2 µm. (***H***) SH-SY5Y cells were incubated with 50 µM CORT or not for 48 h, or with 50 µM CORT in the presence of 10 µM RU486 for 48 h, then subjected to HSV-1 infection for 24 h at MOI=1. HSV-1 strain F viral titer: 5×10^7^ PFU/mL. Expression of gB and ICP27 was detected at 24 h p.i. GAPDH was used as a loading control. Immunoblot was performed in three independent experiments, and representative blotting results were shown. (***I***) Fold change of protein level was calculated using Image J software. One-way ANOVA followed by Tukey's multiple comparisons test was used to assess the statistical significance. Data are presented as means ± SD of three independent experiments. Significances were marked as ^**^P< 0.01 vs. indicated group. (***J***) Viral productions by cells from (***H***) were quantified by plaque assay. One-way ANOVA followed by Tukey's multiple comparisons test was used to assess the statistical significance. Data were presented as mean ± SD of three independent experiments. ^**^P < 0.01 *vs.* indicated group.

Subsequently, CORT was used to induce cell stress in the *in vitro* studies as we previously described 39. We found that CORT treatment at 25 μM, 50 μM for 48 h could increase the replication of HSV-1, as evidenced by significantly increased viral yield in SH-SY5Y cells (Figure 2A). Moreover, the viral yield was significantly higher after 48 h treatment of 50 μM CORT than that of 24 h (Figure 2B-C). Real-time PCR showed significant increases in the levels of viral UL27, UL52 and UL54 transcripts in HSV-1-infected SH-SY5Y cells after CORT treatment (Figure 2D). In addition, significantly increased protein expression of gB was also detected in CORT-pretreated SH-SY5Y cells (Figure 2E-F). To provide further evidence that CORT treatment increases the susceptibility to HSV-1 infection, we performed TEM to determine the ultrastructure of infected SH-SY5Y cells. The results revealed that the number of virions increased under CORT stimulation (Figure 2G). Interestingly, the phenomenon that CORT-induced susceptibility of cells to HSV-1 seemed to be universal, since increased viral yield was also observed in PC12 cells (Figure S1A), HT22 cells (Figure S1B-D) and primary mouse cortical neurons under CORT treatment (Figure S1E-F).

However, RU486 significantly alleviated the increased protein expression of gB and ICP27 (Figure 2H-I), and the viral yield in HSV-1-infected cells under CORT loading (Figure 2J and Figure S1E-F). Together, these findings indicate that CORT increases the susceptibility of cells to HSV-1 *in vitro*.

### CORT activates autophagy

**Figure 3:**
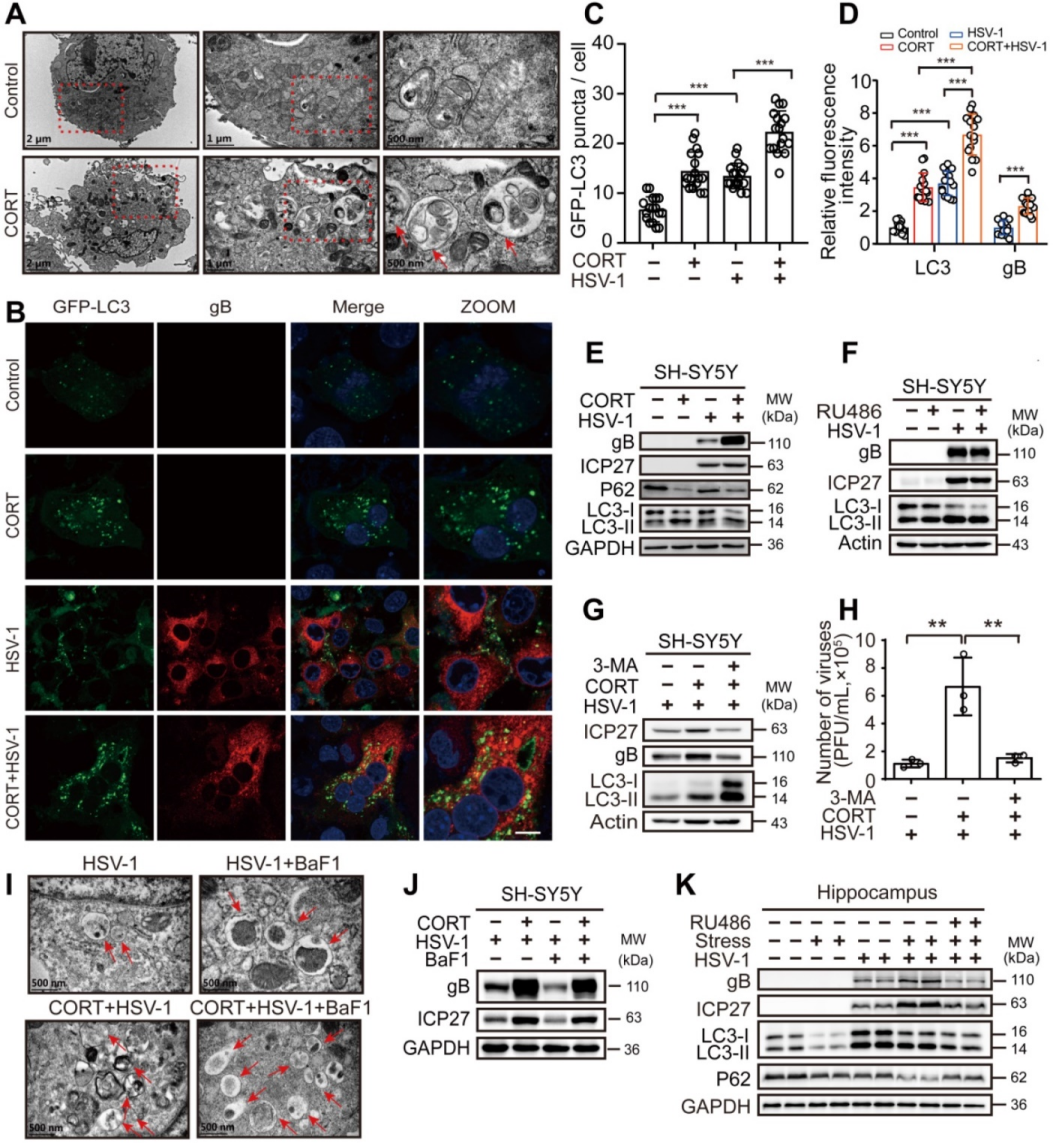
** CORT activates autophagy.** (***A***) SH-SY5Y cells were analyzed by TEM after exposure to 50 µM CORT or not for 48 h. Red dashed frames demarcated the enlarged areas. Red arrows indicated autophagosomes or autophagosome-like structures. Scale bars, 2 µm, 1 µm, 500 nm. (***B***) SH-SY5Y cells were transfected with GFP-LC3. At 12 h after transfection, cells were treated with 50 µM CORT or not for 48 h, followed by HSV-1 infection (MOI=1) for another 24 h. HSV-1 strain F viral titer: 5×10^7^ PFU/mL. Cells were fixed with 4% paraformaldehyde and stained with anti-gB (red). Nuclei were labeled with DAPI (blue). Scale bar, 10 µm. (***C***) GFP-LC3 puncta in (***B***) were counted in randomly selected fields by using Image J software (National Institutes of Health, Bethesda, MD, USA). Non-parametric Wilcoxon Mann-Whiney U-test was applied to assess the statistical significance. Data were presented as mean ± SD. ^***^P < 0.001 *vs.* indicated group. (***D***) The mean fluorescence intensity of LC3 and gB of (***B***) was quantified in randomly selected fields by using Image J software. Non-parametric Wilcoxon Mann-Whiney U-test was applied to assess the statistical significance. Data were presented as mean ± SD. Significances were marked as ^***^P < 0.001 *vs*. indicated group. (***E***) SH-SY5Y cells were treated with 50 µM CORT or not for 48 h, followed by HSV-1 infection (MOI=1) for 24 h. HSV-1 strain F viral titer: 5×10^7^ PFU/mL. Immunoblot analysis was performed to detect gB, ICP27, P62 and LC3. (***F***) SH-SY5Y cells were treated with 10 µM RU486 for 48 h, then subjected to HSV-1 infection for 24 h at MOI=1. HSV-1 strain F viral titer: 5×10^7^ PFU/mL. Protein expression of gB, ICP27 and LC3 was detected. (***G***) SH-SY5Y cells were divided into three groups: HSV-1, CORT + HSV-1 and 3-MA + CORT + HSV-1 (10 mM 3-MA, 24 h and 50 µM CORT, 48 h). Cells were harvested at 24 h p.i., and whole-cell lysates were prepared for immunoblot analysis. HSV-1 strain F viral titer: 5×10^7^ PFU/mL. (***H***) Production of viruses by cells in (***G***) was quantified by plaque assay. One-way ANOVA followed by Tukey's multiple comparisons test was used to assess the statistical significance. Data were presented as mean ± SD of three biological samples. ^**^P < 0.01 *vs.* indicated group. (***I***) SH-SY5Y cells were exposed to 50 µM CORT or not for 48 h, then infected with HSV-1 (MOI=1) for another 24 h. HSV-1 strain F viral titer: 5×10^7^ PFU/mL. Cells were analyzed by TEM in the absence or presence of 50 nM BaF1. Red arrows indicated autophagosomes or autophagosome-like structures. Scale bar, 500 nm. (***J***) SH-SY5Y cells were exposed to 50 µM CORT or not for 48 h, then infected with HSV-1 (MOI=1) for another 24 h. HSV-1 strain F viral titer: 5×10^7^ PFU/mL. Cells were harvested in the absence or presence of 50 nM BaF1. Immunoblot analysis was performed in two independent experiments to analyze the expression of the indicated proteins, and representative blotting results were shown. (***K***) Proteins were extracted from mouse hippocampus for immunoblot analysis with the indicated antibodies. n=2 mice.

Autophagy functions broadly in systemic immunity 42. As reported previously, autophagy exerts both antiviral and proviral effects depending on virus type, cell type and cellular environment 43-46. Interestingly, autolysosome-like structures were observed by TEM in cells treated with CORT (Figure 3A). Accordingly, confocal microscopy revealed increased autophagosomes formation in CORT treated cells, as evidenced by significantly increased GFP-LC3 puncta and relative fluorescence intensity of LC3, together with increased transition of LC3-I to LC3-II and decreased expression of P62. Interestingly, HSV-1 also induced autophagy, which was even increased when pre-treated with CORT (Figure 3B-E). Notably, HSV-1 was more actively replicated under CORT treatment, as indicated by increased fluorescence intensity of gB (Figure 3B-D) and increased expression of ICP27 and gB (Figure 3E). Moreover, the autophagic activity and viral protein expression in CORT pre-treated cells at different time points during HSV-1 infection were also analyzed. We can clearly see that HSV-1 infection gradually increased the expression of gB protein and the transition of LC3-I to LC3-II, which were augmented by CORT treatment up to 12 h. However, the induction of autophagy is an event that can be saturated. The formation of autophagosomes is limited to ensure that a large number of accumulated autophagosomes can be fully degraded. This can explain why LC3-II/I was not significantly different between CORT group and control group at 24 h of HSV-1 infection. Nevertheless, at this time point, the expression of P62 was decreased in the CORT group, suggesting an intact autophagic flux in this state (Figure S2). Noteworthy, RU486 alone did not have any effect on HSV-1 replication, nor did it change autophagy (Figure 3F). Subsequently, we found that treatment of cells with 3-methyladenine (3-MA), a widely used autophagy inhibitor, resulted in decreased viral protein expression and viral replication in SH-SY5Y cells treated with CORT (Figure 3G-H). The effect of BaF1, an inhibitor of lysosomal acidification, was also tested in cells treated with HSV-1 and CORT + HSV-1. As a result, CORT induced a dramatic increase of autophagic flux (Figure 3I, Figure S3A-D). Interestingly, BaF1 treatment inhibited viral protein expression (Figure 3J). Additionally, CORT promoted HSV-1 replication not only in SH-SY5Y cells, PC12 cells, HT22 cell and primary mouse cortical neurons but also in NRK cells and GFP-LC3-LAMP1-Cherry-NRK cells. BaF1 reduced CORT-induced expression of gB, albeit only moderately (Figure S3A, C and D).

*In vivo*, ICP27 and gB protein expression in mouse hippocampus of the model group increased compared with the virus only group. RU486 treatment prevented the increased expression of ICP27 and gB in the model group. Interestingly, restrained stress also activated autophagy, as evidenced by increased transition of LC3-I to LC3-II and decreased expression of P62 (Figure 3K). Thus, these data provide further evidence that CORT-induced susceptibility to HSV-1 infection is associated with activated autophagy.

### CORT induces autophagy in an ULK1-independent manner

**Figure 4:**
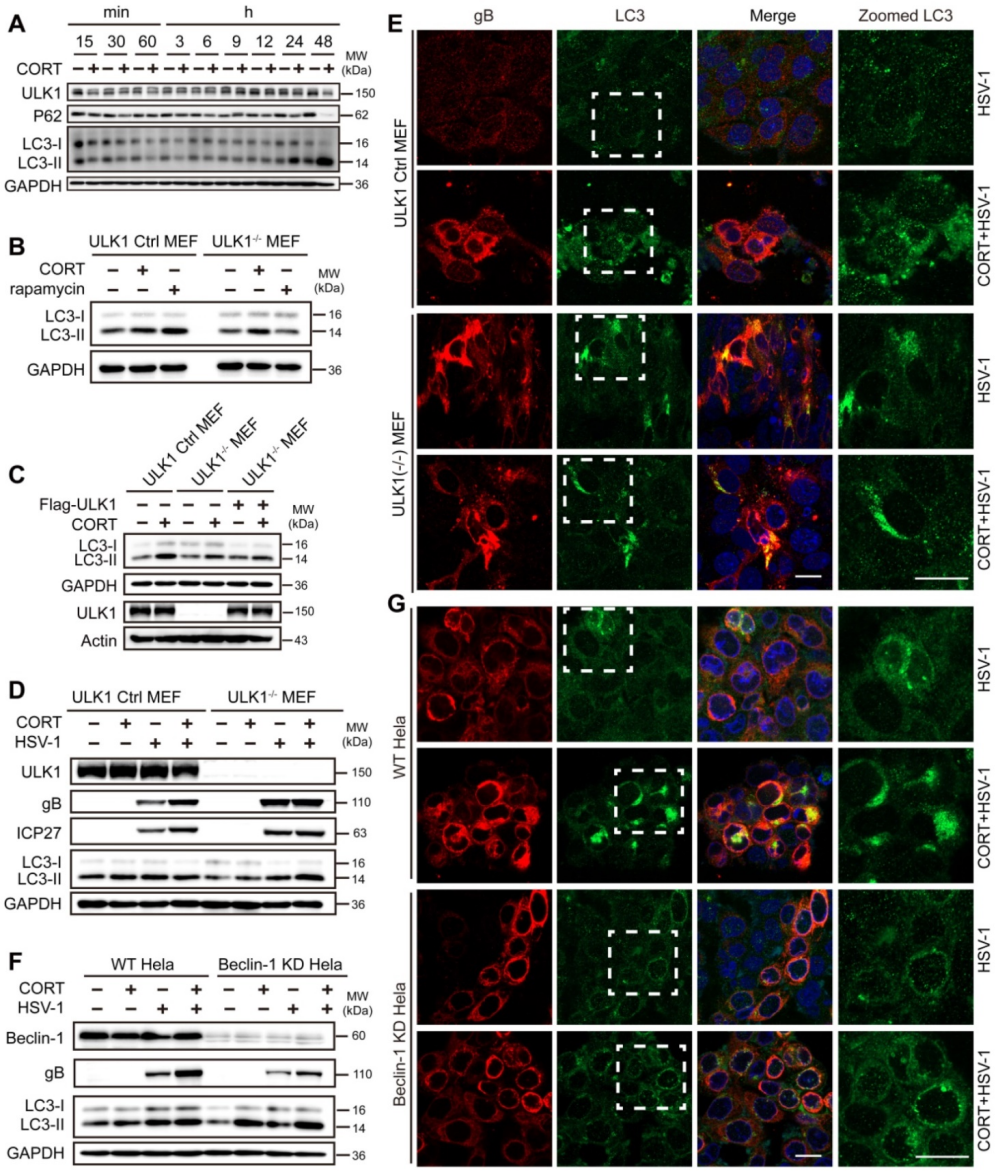
** CORT induces autophagy in an ULK1-independent manner.** (***A***) Time-course measurement of CORT-induced autophagy. SH-SY5Y cells were incubated with or without CORT (50 µM) for the indicated time. Whole-cell lysates were analyzed by immunoblot analysis. (***B***) ULK1 Ctrl MEF (ULK1 control MEFs) and ULK1^-/-^ MEF (ULK1 KO MEFs) were individually treated with 50 µM CORT for 48 h, or exposed to 100 nM rapamycin for 12 h. The protein expression of LC3 was detected by immunoblot analysis. Immunoblot was performed in four independent experiments, and representative blotting results were shown. (***C***) ULK1 Ctrl MEF and ULK1^-/-^ MEF were transfected with Flag-ULK1 or not, followed by the treatment of 50 µM CORT for 48 h or not. Cell lysates were prepared for immunoblot analysis with the indicated antibodies. Immunoblot analysis was performed in two independent experiments, and representative blotting results were shown. (***D***) ULK1 Ctrl MEF and ULK1^-/-^ MEF were exposed to 50 µM CORT or not for 48 h, followed by HSV-1 infection (MOI=1) for 24 h. HSV-1 strain F viral titer: 5×10^7^ PFU/mL. Cells were harvested at 24 h p.i. Cell lysates were prepared for immunoblot analysis. Immunoblot was performed in two independent experiments, and representative blotting results were shown. (***E***) ULK1 Ctrl MEFs and ULK1^-/-^ MEFs were treated as described in (***D***), then cells were fixed with 4% paraformaldehyde and stained with anti-gB (red), anti-LC3 (green) and DAPI (blue) for immunofluorescence microscopy. Scale bars, 20 µm. (***F***) WT HeLa (wild-type HeLa) cells and Beclin-1 KD HeLa (Beclin-1 knockdown HeLa) cells were exposed to 50 µM CORT or not for 48 h, followed by HSV-1 infection (MOI=1) for 24 h. HSV-1 strain F viral titer: 5×10^7^ PFU/mL. Whole cell lysates were collected for immunoblot analysis. Immunoblot was performed in two independent experiments, and representative blotting results were shown. (***G***) WT HeLa cells and Beclin-1 KD HeLa cells were treated as described in (***F***), then cells were fixed with 4% paraformaldehyde and stained with anti-gB (red), anti-LC3 (green) and DAPI (blue). Scale bars, 20 µm.

Time-course experiments showed an increased ratio of LC3-II/I together with an increased degradation of P62 in CORT-treated SH-SY5Y cells, which implies obvious autophagy induction. Nevertheless, ULK1 protein did not increase following CORT treatment, which suggested that ULK1 might not be associated with CORT-induced autophagy (Figure 4A, Figure S4A-B). Furthermore, protein expression of Beclin-1 and ATG5 was also not obviously changed in the presence of CORT (Figure S4A-B). Hence, we used ULK1^-/-^ MEFs and Beclin-1 KD HeLa cells to investigate the molecular basis of autophagy induced by CORT. ULK1 is a serine/threonine kinase that initiates autophagy in response to nutrient deprivation 47. Rapamycin upregulates autophagy by inhibiting the mTOR-ULK1 pathway 48. To investigate whether CORT-induced autophagy is ULK1-independent, we have introduced rapamycin as an ULK1-dependent autophagy-inducing positive control. The results showed that CORT could induce autophagy in both ULK1 Ctrl MEFs and ULK1^-/-^ MEFs. However, rapamycin-induced autophagy could only be observed in ULK1 Ctrl MEFs (Figure 4B). To further understand the role of ULK1 in CORT-induced autophagy, we restored the expression of ULK1 by transfecting Flag-ULK1 in ULK1^-/-^ MEFs. As shown in Figure 4C, the presence or absence of ULK1 did not affect CORT-induced autophagy.

Notably, there was no obvious difference in the protein levels of ULK1 and Beclin-1 in wild-type cells, ULK1^-/-^ MEFs or Beclin-1 KD HeLa cells between control group and CORT group (Figure 4D-F), which suggested that ULK1 and Beclin-1 did not respond during CORT-induced autophagy. Furthermore, knockout of ULK1 seemed not to inhibit gB protein expression and the transition of LC3-I to LC3-II in the presence of CORT (Figure 4D-E and Figure S5A-B), even if the expression of gB and ICP27 was not obviously increased in CORT-treated ULK1^-/-^ MEFs (Figure 4D-E and Figure S5B). Unlike knockout of ULK1, knockdown of Beclin-1 clearly inhibited gB protein expression; however, this inhibition did not prevent the increase of gB protein expression in Beclin-1 KD HeLa cells under CORT treatment (Figure 4F-G and Figure S5D).

In addition, LC3 aggregation was not reduced in Beclin-1 KD HeLa cells under CORT treatment (Figure 4F-G and Figure S5C-D). Taken together, these data suggest that CORT induced-autophagy is dependent on LC3 lipidation but does not require ULK1.

### CORT-induced HSV-1 susceptibility is associated with the reduction in the level of PML protein

**Figure 5:**
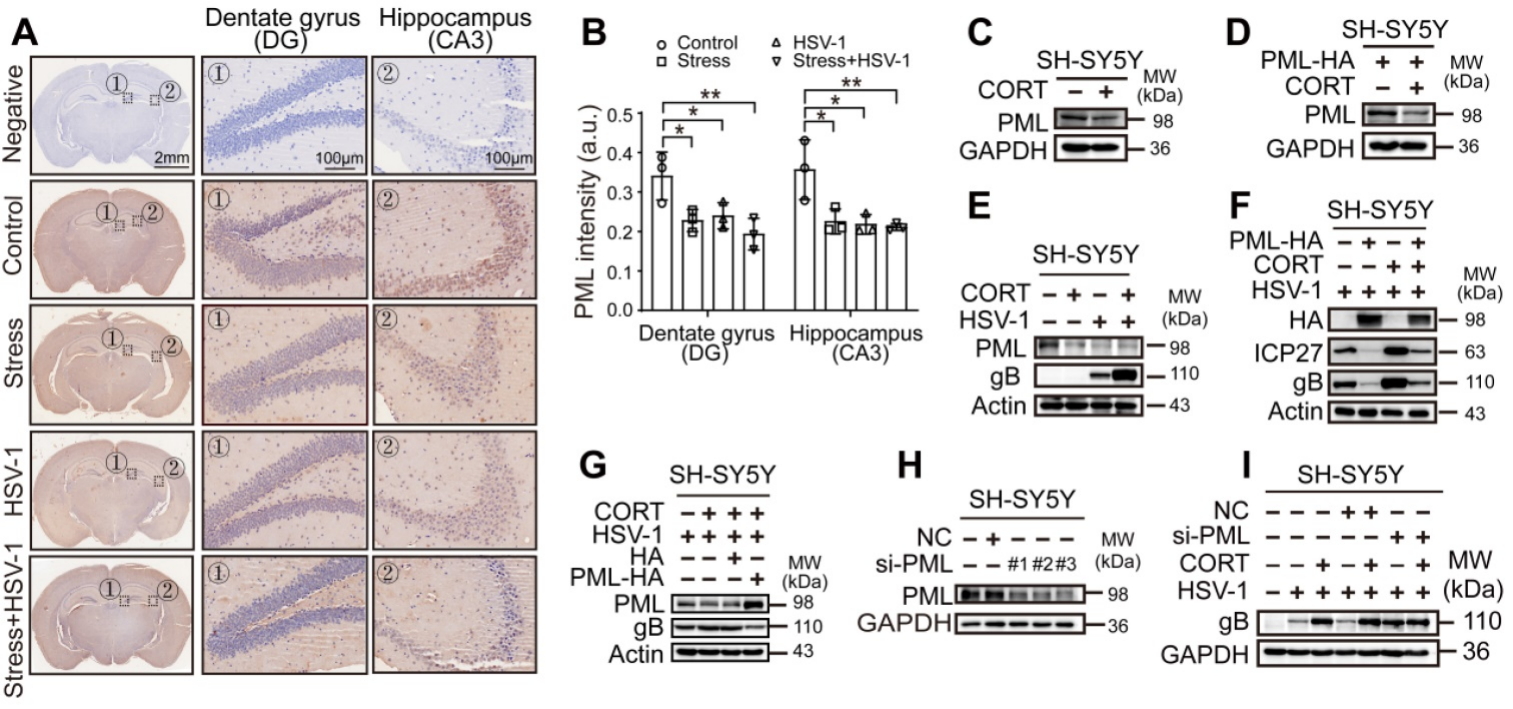
** CORT-induced HSV-1 susceptibility is associated with the reduced level of PML protein.** (***A***) Representative IHC staining of PML in the hippocampus. The black dotted boxes (1 and 2) indicated the areas that were enlarged in the images labeled 1 (Dentate gyrus, DG) and 2 (Hippocampus, CA3). Scale bars, 2 mm, 100 µm. n=3 mice. (***B***) Mean immunohistochemistry signal intensity (arbitrary units, a.u.) of PML in DG and CA3. Data were analyzed by one-way ANOVA and Dunnett's multiple comparisons test was used to assess the statistical significance. Data were presented as mean ± SD. n=3 mice. ^*^P < 0.05, ^**^P < 0.01 *vs.* indicated group. (***C***) Endogenous protein expression of PML in SH-SY5Y cells under CORT stimuli. Immunoblot was performed in two independent experiments, and representative blotting results were shown. (***D***) Exogenous protein expression of PML. SH-SY5Y cells were transfected with PML-HA for 12 h, then incubated with 50 µM CORT or not for 48 h. Cell lysates were prepared for immunoblot analysis. Immunoblot was performed in two independent experiments, and representative blotting results were shown. (***E***) SH-SY5Y cells were treated with 50 µM CORT or not for 48 h, followed by HSV-1 infection (MOI=1) for 24 h. HSV-1 strain F viral titer: 5×10^7^ PFU/mL. Expression of the indicated proteins was assessed by immunoblot analysis. (***F***) SH-SY5Y cells were transfected with PML-HA or not for 12 h. Cells were then cultured in the absence or presence of 50 µM CORT for 48 h, then infected with HSV-1 (MOI=1). Whole-cell lysates were prepared for immunoblot analysis at 24 h p.i. HSV-1 strain F viral titer: 5×10^7^ PFU/mL. (***G***) SH-SY5Y cells were divided into four groups: HSV-1, CORT + HSV-1 (cells were treated with 50 µM CORT for 48 h before HSV-1 infection), CORT + HSV-1 + HA (cells were treated with 50 µM CORT for 48 h, then infected with HSV-1, then transfected with HA plasmid), and CORT + HSV-1 + PML-HA (cells were treated with 50 µM CORT for 48 h, then infected with HSV-1, then transfected with PML-HA plasmid). In all groups involving HSV-1 infection, the HSV-1 infection time was 24 h with a MOI of 1. HSV-1 strain F viral titer: 5×10^7^ PFU/mL. Specific proteins were analyzed by immunoblotting with the indicated antibodies. (***H***) Endogenous PML protein levels in SH-SY5Y cells were determined by immunoblotting analysis, after transfection of siRNA against PML for 24 h. Immunoblot was performed in four independent experiments, and representative blotting results were shown. (***I***) The expression of gB in PML-silenced SH-SY5Y cell by immunoblotting analysis. SH-SY5Y cells were transfected with siRNA specific for PML, and treated with 50 µM CORT or not for 48 h, then infected with HSV-1 (MOI=1). HSV-1 strain F viral titer: 5×10^7^ PFU/mL. Immunoblot was performed in three independent experiments, and representative blotting results were shown.

We next sought to dissect the precise mechanism by which CORT-induced autophagy participated in the regulation of HSV-1 infection. It has been reported that PML plays an important role in both innate immunity and atypical intrinsic immunity 49, 50. Moreover, a number of lines of evidences support an important role of PML in repressing HSV-1 infection 28, 29. Consequently, we tested the protein expression of PML in the brains of mice subjected to restraint stress and HSV-1 infection. Interestingly, IHC staining demonstrated a relatively lower level of PML expression in the dentate gyrus (DG) and hippocampus (CA3) of both HSV-1 infected and restraint stress-overloaded group (Figure 5A-B).

*In vitro*, both endogenous PML and exogenously overexpressed PML decreased under CORT stimulus (Figure 5C-D, Figure S6A-D). Notably, the protein expression of PML was also decreased in either CORT-overloaded or HSV-1-infected SH-SY5Y cells, HT22 cells and TM4 cells. The combination of CORT with HSV-1 infection induced even further reduction of PML protein, and this reduction was associated with increased viral replication as evidenced by increased expression of viral proteins (Figure 5E, Figure S6E-F). In light of these data, we hypothesized that PML failed to prevent the replication of virions in the presence of stress stimuli due to decreased protein levels of PML. To verify this hypothesis, we transiently transfected SH-SY5Y cells with PML-HA before administration of CORT and infection with HSV-1. A striking decrease in protein expression of ICP27 and gB was observed in PML-overexpressing cells. However, this inhibition was reversed by CORT treatment (Figure 5F). To further validate this observation, SH-SY5Y cells were incubated with CORT or not, and infected with HSV-1. HA and PML-HA were then transfected into the cells after infection. We found that PML inhibited the protein expression of gB, even in CORT-pretreated cells (Figure 5G).

Moreover, knockdown of PML by siRNA technique has been performed. The data indicated that three siRNAs specific to PML had good interference effect (Figure 5H). As expected, the protein expression of gB increased in virus-infected SH-SY5Y cells interfered with si-*PML* comparing with NC group and the virus only group, which can be explained with the results that virus dampens PML level. Furthermore, the data revealed that there were no obvious differences of gB protein level between “HSV-1” and “CORT+HSV-1” groups when PML was silenced, which provided direct evidence linking reduction of PML and CORT-induced virus susceptibility (Figure 5I).

### CORT-induced autophagy contributes to the degradation of PML

**Figure 6:**
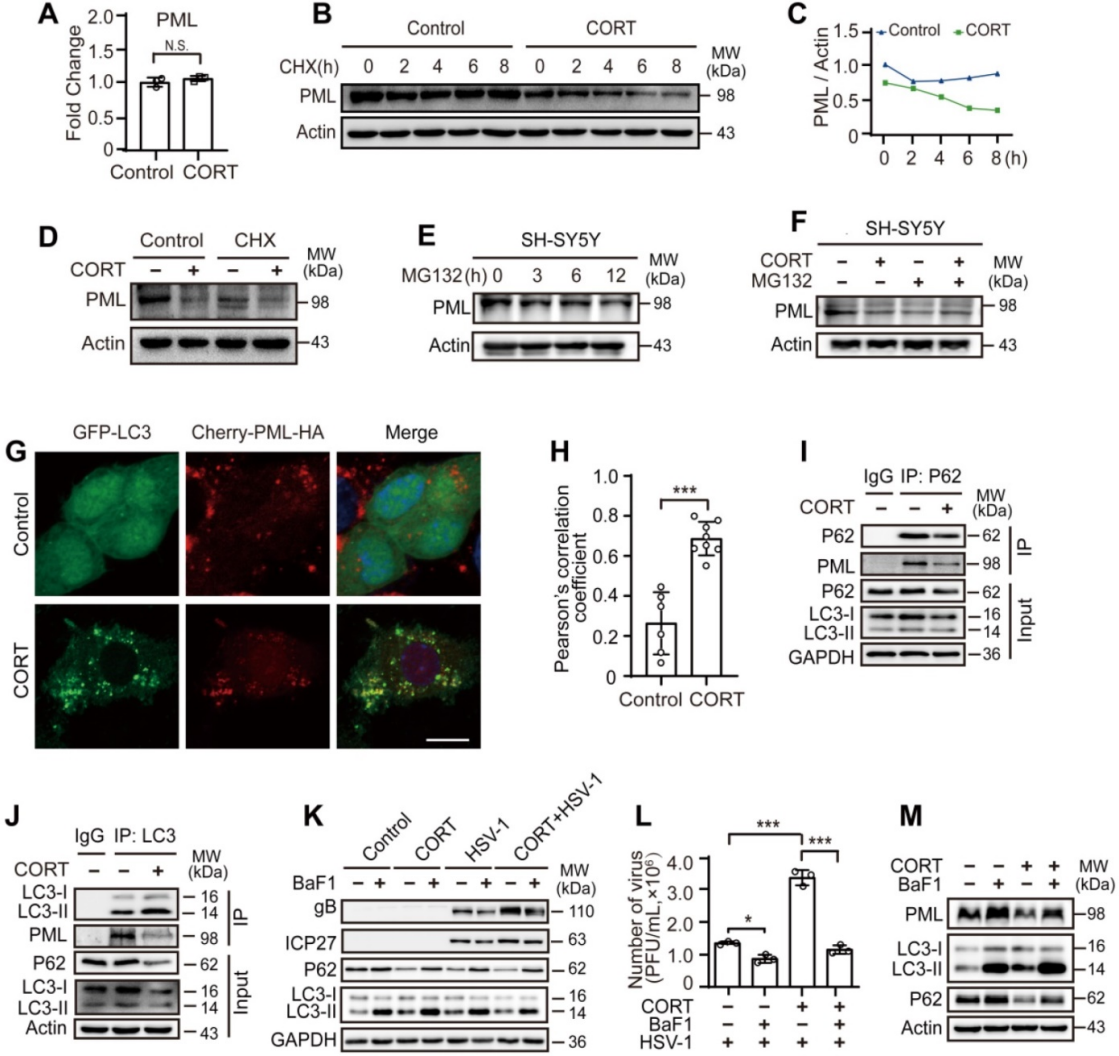
** CORT-induced autophagy contributes to the degradation of PML.** (***A***) Fold change of PML mRNA. SH-SY5Y cells were incubated with 50 µM CORT or not for 48 h. PML mRNA was detected by real-time PCR. A non-parametric Wilcoxon Mann-Whiney U-test was used for statistical analyses. Data were presented as mean ± SD of three biological samples. N.S. = not significant. (***B***) SH-SY5Y cells were incubated with 50 µM CORT or not for 48 h, then exposed to 100 µM CHX for the indicated times. Expression of PML was assessed by immunoblot analysis. (***C***) The PML/Actin ratio was calculated by Image J. (***D***) After 6 h incubation with CHX (100 µM), SH-SY5Y cells were treated with or without 50 µM CORT for 48 h. Immunoblot analysis was used to detect the expression of PML. (***E***) SH-SY5Y cells were treated with 10 µM MG132 for 0, 3, 6, 12 h. Total cellular protein was extracted and the expression of PML was detected. (***F***) SH-SY5Y cells were divided into four groups: Control, CORT (50 µM, 48 h), MG132 (10 µM, 24 h), CORT + MG132 (cells were treated with 50 µM CORT for 24 h before simultaneously incubation with 10 µM MG132 for another 24 h). The expression of PML was detected. (***G***) Co-location of PML and LC3. SH-SY5Y cells were co-transfected with GFP-LC3 and Cherry-PML-HA for 12 h. Cells were cultured in the absence or presence of 50 µM CORT for 48 h. Cells were fixed with 4% paraformaldehyde and stained with DAPI (blue). Scale bar, 10 µm. (***H***) Pearson's correlation coefficient was calculated by using Image J software to indicate the co-localization of LC3 and PML in (***G***). Non-parametric Wilcoxon Mann-Whiney U-test was applied to assess the statistical significance. Data were presented as mean ± SD. ^***^P < 0.001 *vs.* indicated group. (***I-J***) Co-IP of PML with LC3 and P62. SH-SY5Y cells were treated with 50 µM CORT or not for 48 h, and were subjected to co-IP with an anti-P62 antibody or an anti-LC3 antibody. (***K***) SH-SY5Y cells were exposed to 50 µM CORT for 48 h, then infected with HSV-1 (MOI=1) for another 24 h. HSV-1 strain F viral titer: 5×10^7^ PFU/mL. Cells were harvested in the absence or presence of 50 nM BaF1. Immunoblot analysis was performed to analyze the expression of the indicated proteins. (***L***) Viral productions by cells in (***K***) were quantified by plaque assay. One-way ANOVA followed by Tukey's multiple comparisons test was used to assess the statistical significance. Data were shown as mean ± SD of three biological samples. ^*^P < 0.05, ^***^P < 0.001 *vs*. indicated group. (***M***) The expression of PML, LC3 and P62 was detected by immunoblot analysis. SH-SY5Y cells were treated with 50 µM CORT or not for 48 h. Cells were harvested in the absence or presence of 50 nM BaF1.

We next addressed how PML was degraded by CORT treatment. First, the mRNA level of PML under CORT treatment was detected. We found that PML was not regulated by CORT at transcriptional level (Figure 6A). Mechanistically, eukaryotic cells use two major systems to orchestrate protein degradation: the ubiquitin-proteasome system (UPS) and the lysosome pathway 51, 52. To determine whether the decreased PML was due to the protein turn-over rate or stability, SH-SY5Y cells were treated with CHX that inhibits translational elongation. Interestingly, CORT accelerated the degradation of PML in the presence of CHX (Figure 6B-6D). Furthermore, the protein expression of PML did not change in cells treated with MG132, a proteasome inhibitor (Figure 6E, Figure S7A-B). Moreover, MG132 neither increased endogenous PML protein levels, nor prevented PML protein degradation induced by CORT treatment (Figure 6F, Figure S7C), indicating that CORT-driven PML degradation is independent of proteasome pathway.

Since CORT induced autophagy (Figures 3-4, Figures S2-5), we speculated that autophagy might be engaged in the degradation of PML. Endogenous fluorescence staining showed significant decrease of PML puncta in the nucleus and relative lower fluorescence intensity of PML in cells exposed to CORT (Figure S8A-C). Furthermore, immunofluorescence analysis confirmed that CORT treatment significantly increased the co-localization of PML and LC3 (Figure 6G-H). For PML, there are several isoforms with different subcellular localization 53. The plasmid (Cherry-PML [NM_033238.2] */HA) we constructed is PML-I, also known as TRIM19 alpha, which is the most abundant (but perhaps least studied) isoform. In addition to the nuclear localization signal (NLS) present in all PML isoforms, PML-I harbors a nuclear export signal (NES) that allows shuttling of all isoforms between the two compartments through heterodimer formation 54-56. Due to the nuclear export signal, PML-I forms some cytoplasmic PML-labeled bodies.

Previous work on acute promyelocytic leukemia (APL) showed that autophagy contributed to the basal turnover and therapy-induced degradation of the oncoprotein PML-RARα, a fusion protein of PML and retinoic acid receptor-α 57, 58. However, whether PML is degraded by CORT-induced autophagy is still unknown. Accordingly, we performed co-immunoprecipitation (co-IP) of PML and P62. Indeed, PML interacted with P62, and the protein expression of P62 was decreased in CORT-treated cells (Figure 6I). As previously reported, LC3-binding proteins, including P62, contain a short hydrophobic WxxL LC3 interacting region (LIR) 59, 60. Since we have observed the co-localization between PML and LC3 (Figure 6G), we wondered whether PML could also interact with LC3. Co-IP demonstrated that PML interacted with LC3 (Figure 6J), in addition to P62 (Figure 6I). These results indicate that PML is a target of CORT-induced autophagy.

Additionally, reduced viral replication was observed in the presence of BaF1 (Figure 6K-L), accompanied by accumulated PML protein (Figure 6M and Figure S8D). To further confirm the importance of autophagy in these molecular events, we depleted LC3 by RNAi and then monitored the susceptibility of the cells to HSV-1 infection in the presence or absence of CORT by detecting the expression of gB and ICP27. Interestingly, the expression of viral proteins was greatly reduced in LC3-depleted cells compared to the NC group. The degradation of PML by CORT-induced autophagy was prevented in cells with RNAi knockdown of LC3 (Figure S8E). In view of the antiviral effect of PML in intracellular immunity and the autophagic degradation of PML under stressful stimuli, our data strongly suggests that restraint stress/CORT-induced autophagy facilitates viral replication rather than eliminates it, and clarifies the molecular mechanisms underlying psychological stress-induced HSV-1 susceptibility.

## Discussion

Previous studies have shown that stress is correlated with HSV-1 infection 6, 61. The capabilities of the immune system are diminished by chronic stresses, which affect the sympathetic-medullary (SAM) system, hypothalamic-pituitary-adrenocortical (HPAC) system, and other endocrine systems 62. Our results are consistent with this concept and indicate that CORT can down-regulate the expression of IFNβ (Figure S9A-D), chemokines (Figure S9A), and interferon-stimulated genes (ISGs) (Figure S9A). Cyclic GMP-AMP Synthase (cGAS), stimulator of interferon genes (STING), and TANK Binding Kinase 1 (TBK1) have been reported to be involved in HSV-1-induced autophagy 63-66. We therefore examined the protein expression of cGAS, STING, TBK1 and phospho-TBK1 under CORT treatment. Interestingly, CORT decreased the protein expression of cGAS and STING, but had no effect on TBK1 and phospho-TBK1 (Figure S9C-D). Consequently, whether cGAS, STING and TBK1 are involved in CORT-induced autophagy remains to be further determined.

Nerve cells are non-renewable, and therefore their immune response is more dependent on nondestructive innate defense and intrinsic immunity 9. HSV-1 infection triggers a rapid activation of the host innate immunity. Apart from the type I IFN signal pathway, PML, an intrinsic host gene product, also restricts HSV-1 infection 23, 24. It has been hypothesized that PML represses the transcription of the virus by enveloping viral genomes and limiting their interactions with host factors that stimulate viral transcription 28. However, HSV-1 seems to have evolved multiple strategies to evade the host immune response, establishing a life-long latent infection in neurons 67. ICP0, one of the five immediate early (IE) proteins, facilitates viral gene expression and impairs the host's antiviral responses to infection through directing the attachment of ubiquitin molecules to target proteins for degradation by proteasome 68. During HSV-1 infection, viral protein ICP0 induces the degradation of PML, thereby disrupting nuclear domain 10 (ND10) 30, 31. It has been uncovered that a portion of the C terminus (amino acids 633 to 767), the nuclear localization signal (NLS) and two overlapping regions within the central N-terminal portion of ICP0 (residues 212 to 311) facilitate its dissociation and degradation of PML 69. In our study, we also observed decreased expression of PML protein after HSV-1 infection (Figure 5, Figures S6-8). Furthermore, we have checked the protein expression of ICP0 in HSV-1 infected cells exposed to CORT. Notably, ICP0 protein expression was not affected by CORT, suggesting that ICP0 did not contribute to the decrease in PML under CORT stimulus (Figure S10).

Previous studies have made pioneering efforts to elucidate the effects of autophagy on viral infection. In fact, autophagy acts as a double-edged sword in viral infection, exerting both antiviral and proviral effects depending on virus type, cell type and cellular environment 43-46. In our study, CORT, a natural stress hormone, was able to activate autophagy and thus resulted in the degradation of PML in response to HSV-1 infection. This interesting disclosure was indeed supported by previously reported evidences that PML interacted with the autophagic cargo receptor P62 and the autophagic effector protein LC3 59, 60. Overall, in our experiment setting, autophagy induced by CORT caused a productive virus infection, rather than the elimination of HSV-1.

Amongst the realm of models to study or intervene with the infection of HSV-1, we described the HSV-1 susceptible mice model and cell model, respectively simulated by restraint stress and CORT-overloaded treatment, according to our previously reported researches 38, 39, 70, 71. It should be mentioned that the effects of CORT on HSV-1 infection seemed to be ubiquitous, since CORT treatment significantly increased HSV-1 replication in multiple cells. In addition, CORT-activated autophagy was also observed in a variety of cells.

Here, we have shown that CORT-induced autophagy contributes to HSV-1 replication, and this activated autophagy does not seem to depend on ULK1 (Figure 4, Figure S4). It has been reported that the immune function of conserved autophagy proteins often reflects a non-canonical form of autophagy (NCA) involved in membrane trafficking events during dsDNA recognition 72, 73. However, NCA might inhibit intracellular pathogen replication 74, 75 or suppress immune reactions 76. Likewise, how CORT signaling activates autophagy pathways and whether CORT-induced autophagy is NCA remain to be further studied.

For decades, the ubiquitin-proteasome pathway and SUMO modification have been implicated as the main mechanisms involved in PML degradation 32-34. It has been shown that autophagy also contributes to degradation of the PML-RARα oncoprotein 57, 58. Overexpression of PML significantly enhances the interaction of PML with LC3. Further research revealed that two LIRs in PML were crucial for the interaction between PML and LC3 77. Our data show that CORT-induced HSV-1 susceptibility is associated with reduced levels of PML protein (Figure 5A-B, E and I, Figure S6E-F), and CORT-induced autophagy contributes to the degradation of PML (Figure 6G-J and M, Figure S8D-E). Endogenous PML staining under CORT revealed a significantly decreased PML puncta in the nucleus and relative lower fluorescence intensity of PML in cells exposed to CORT. In addition, we still observed that cytoplasmic PML co-localized with LC3 in CORT group (Figure S8A). The cytoplasmic PML may be other subtypes of PML, or it may be caused by the translocation of PML-I from the nucleus to the cytoplasm. This is an interesting phenomenon worthy of further exploration. It should also be stressed in particular that our study has several limitations. Whether SUMO modification is involved in the mechanisms of PML degradation induced by CORT is still to be discussed.

**Figure 7:**
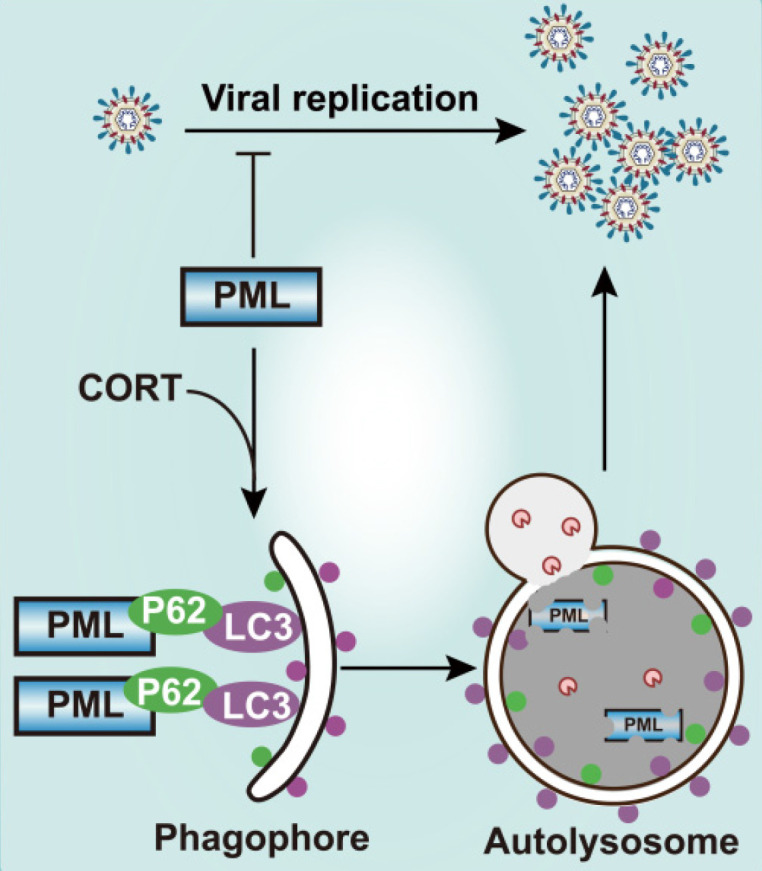
** Schematic diagram summarizing the role of PML in stress-induced autophagy and HSV-1 infection.** Once HSV-1 infection occurs, PML rapidly entraps viral DNA, leading to a blocked viral replication. However, CORT (a stress-induced hormone) activates autophagy and generates autophagosomal membranes carrying LC3 and P62, which bind to PML. The induction of autophagy thus contributes to the degradation of PML, leading to increased susceptibility to HSV-1 under stress.

The crosstalk between PML and autophagy in the process of stress-induced viral susceptibility remains poorly defined. Here we showed that restraint stress caused susceptibility to HSV-1 *in vivo*, and we confirmed that CORT was the mediator of viral susceptibility *in vitro*. As an intrinsic factor that restricts HSV-1 infection, PML rapidly entraps viral DNA, thus blocking viral replication 23. However, our data demonstrates that CORT is able to activate autophagy. Interestingly, PML interacts with the autophagic cargo receptor P62 and the autophagic effector protein LC3. The activation of autophagy by CORT and the interaction of PML with P62 and LC3 thus contribute to the degradation of PML, leading to increased susceptibility to HSV-1 under stress (Figure 7). Therefore, these data suggest a possible mechanism underlying the susceptibility to HSV-1 under stressful conditions which broadens our understanding of the interplay between host and viruses, and a comprehensive understanding of the role of autophagy in viral infection will provide information for future development of innovative drugs against viral infection.

## Supplementary Material

Supplementary figures.

## Figures and Tables

**Table 1:** Primer list for qRT-PCR

Gene	5' primer	3' primer
h-ISG15	TCCTGGTGAGGAATAACAAGGG	GTCAGCCAGAACAGGTCGTC
h-cGAS	GGGAGCCCTGCTGTAACACTTCTTAT	CCTTTGCATGCTTGGGTACAAGGT
h-IRF3	GAGGTGACAGCCTTCTACCG	TGCCTCACGTAGCTCATCAC
h-IFNβ	GATGAACTTTGACATCCCTGAG	TCAACAATAGTCTCATTCCAGC
h-IFNα4	CTGGGTAATAGGAGGGCCTTGA	GGCTTGAGCCTTCTGGAACTGG
h-CXCL9	CCAACCAAGGGACTATCCACC	CCTTCACATCTGCTGAATCTGG
h-CXCL10	TGGCATTCAAGGAGTACCTC	TTGTAGCAATGATCTCAACACG
h-CXCL11	GGCAGATATTGAGAAAGCCTCC	GCCTTGCTTGCTTCGATTTG
h-RNATES	GGCACGCCTCGCTGTCATCCTCA	CTTGATGTGGGCACGGGGCAGTG
h-TNF-α	CCAGACCAAGGTCAACCTCC	CAGACTCGGCAAAGTCGAGA
h-IL-1β	ATGATGGCTTATTACAGTGGCAA	GTCGGAGATTCGTAGCTGGA
h-MX1	GTTTCCGAAGTGGACATCGCA	CTGCACAGGTTGTTCTCAGC
h-IFIT1	TCAGGTCAAGGATAGTCTGGAG	AGGTTGTGTATTCCCACACTGTA
h-IFIT2	GGAGGGAGAAAACTCCTTGGA	GGCCAGTAGGTTGCACATTGT
h-IFIT3	TCAATAAGGAAGTCCCTGATGC	TATGGACAAACCCTCTAAACCA
h-VIPERIN	TGGGTGCTTACACCTGCTG	GAAGTGATAGTTGACGCTGGTT
h-PML	CCTGGATAACGTCTTTTTCGAG	CAGAAGATGTTGTTGGTCTTGC

## Abbreviation

APL: acute promyelocytic leukemia
ATRX: alpha-thalassemia mental retardation X-linked protein
BaF1: bafilomycin A1
cGAS: cyclic GMP-AMP synthase
CHX: cycloheximide
co-IP: co-immunoprecipitation
CORT: corticosterone
Daxx: death domain associated protein
E gene: early gene
GFAP: glial fibrillary acidic protein
GR: glucocorticoid receptor
H&E staining: hematoxylin-eosin staining
HPAC system: hypothalamic-pituitary-adrenocortical system
HSE: HSV-1 encephalitis
HSV-1: herpes simplex virus type 1
IE gene: immediate early gene
IF: immunofluorescence
IFNs: interferons
IHC: immunohistochemistry
ISGs: interferon-stimulated genes
L gene: late gene
LIR: LC3 interacting region
NCA: non-canonical form of autophagy
ND10: nuclear domain 10
PKR: protein kinase R
PML: promyelocytic leukemia
PML-NBs: promyelocytic leukemia protein-nuclear bodies
SAM system: sympathetic-medullary system
STING: stimulator of interferon genes
TBK1: TANK binding kinase 1
TEM: transmission electron microscopy
