# Supplementary material for: Autophagic degradation of PML promotes susceptibility to HSV-1 by stress-induced corticosterone
Source: Theranostics. 2020 Jul 11;10(20):9032–49. doi: 10.7150/thno.46921 (PMC7415815; doi:10.7150/thno.46921)

Figure S1. CORT increases the susceptibility of cells to HSV-1. (*A*) Viral productions of PC12 cells were quantified by plaque assay. PC12 cells were subjected to CORT according to the indicated concentrations for 48 h, then cells were infected with HSV-1 (MOI=1) and harvested at 24 h p.i.. HSV-1 strain F viral titer: 5×10^7 PFU/mL. Data were analyzed by one-way ANOVA and Dunnett's multiple comparisons test was used to assess the statistical significance. Data were presented as mean ± SD of three independent experiments. $^{*}P < 0.05$, $^{**}P < 0.01$ *vs*. the HSV-1 group. (***B***, ***C***) HT22 cells were incubated with 50 μM CORT or not for 48 h, then cells were infected with HSV-1 (MOI=1) and harvested at 24 h p.i.. HSV-1 strain F viral titer: 5×10^7 PFU/mL. Viral productions were quantified by plaque assay. A non-parametric Wilcoxon Mann-Whiney U-test was used for statistical analyses. Data were presented as mean ± SD of three independent experiments. $^{**}P < 0.01$ *vs.* indicated group. (***D***) Immunoblot analysis of gB and Actin. HT22 cells were incubated with 50 μM CORT or not for 48 h, and then were infected with HSV-1 (MOI=1) and harvested at 24 h p.i.. HSV-1 strain F viral titer: 5×10^7 PFU/mL. (***E***) Primary mouse cortical neurons were subjected to 10 μM CORT or not for 48 h, or subjected to 10 μM CORT in the presence of RU486 for 48 h, followed by HSV-1-EGFP infection at MOI=1. HSV-1-EGFP infection was observed by flow cytometry analysis at 24 h p.i. based on the EGFP signal. HSV-1-EGFP viral titer: 1×10^8 PFU/mL. (***F***) Viral productions in (***E***) were quantified. One-way ANOVA followed by Tukey's multiple comparisons test was used to assess the statistical significance. Data were presented as mean ± SD of three biological samples. $^{***}P < 0.001$ *vs*. the HSV-1 group. $^{\#\#\#}P < 0.001$ *vs*. CORT + HSV-1 group.

Figure S2. CORT activates autophagy in SH-SY5Y cells. SH-SY5Y cells were treated with 50 μM CORT or not for 48 h, followed by HSV-1 infection for the indicated time. HSV-1 strain F viral titer: 5×10^7 PFU/mL.

Figure S3. CORT activates autophagy in GFP-LC3-LAMP1-cherry-NRK cells and NRK cells. (*A*) Immunofluorescence staining of gB, LC3 and LAMP1. GFP-LC3-LAMP1-cherry-NRK cells were subjected to 50 μM CORT or not for 48 h, followed by HSV-1 infection (MOI=1) for 24 h. HSV-1 strain F viral titer: 5×10^7 PFU/mL. Cells were fixed with 4% paraformaldehyde at 24 h p.i., and stained with anti-gB (blue). Merge 1 referred to superimposed channels of GFP-LC3 and LAMP1-cherry. Merge 2 showed superimposed channels of GFP-LC3 and gB. Merge 3 demonstrated superimposed channels of LAMP1-cherry and gB. Merge 4 indicated superposition of the above three channels. Scale bar, 10 μm. (***B***) GFP-LC3 puncta in (***A***) were counted in randomly selected fields by using Image J software. Non-parametric Wilcoxon Mann-Whiney U-test was applied to assess the statistical significance. Data were presented as mean ± SD. $^{**}P < 0.01$, $^{***}P < 0.001$ *vs.* indicated group. (***C***) The mean fluorescence intensity

of Lamp1, LC3 and gB in (***A***) was semi-quantitatively measured by using Image J software. Non-parametric Wilcoxon Mann-Whiney U-test was applied to assess the statistical significance. Data were presented as mean ± SD. Significances were marked as $^{*}P < 0.05$, $^{**}P < 0.01$, $^{***}P < 0.001$ *vs*. indicated group. N.S. = not significant. (***D***) Immunoblot analysis of gB, P62 and LC3. NRK cells were exposed to 50 μM CORT or not for 48 h, followed by HSV-1 infection for another 24 h. HSV-1 strain F viral titer: 5×10^{7} PFU/mL. Cells were harvested in the absence or presence of 50 nM BaF1 and then lysed for immunoblot analysis. s.e, short exposure; l.e, long exposure.

Figure S4. CORT activates autophagy in an ULK1-independent manner. (***A***) SH-SY5Y cells were treated with CORT (50 μM) or not for 48 h, and the levels of specific proteins were determined by immunoblot analysis. Immunoblot was performed in three independent experiments, and representative blotting results were shown. (***B***) Fold change of protein level was calculated by using Image J software. A non-parametric Wilcoxon Mann-Whiney U-test was used for statistical analyses. Data were presented as mean ± SD of three independent experiments. $^{**}P < 0.01$ *vs.* indicated group. N.S. = not significant.

Figure S5. CORT induces autophagy in an ULK1-independent manner. (***A***) GFP-LC3 puncta were counted in randomly selected fields by using Image J software. Non-parametric Wilcoxon Mann-Whiney U-test was applied to assess the statistical significance. Data were presented as mean ± SD. $^{**}P < 0.01$, $^{***}P < 0.001$ *vs.* indicated group. (***B***) The mean fluorescence intensity of gB and LC3 in ULK1 Ctrl MEFs and $ULK^{-/-}$ MEFs was quantified in randomly selected fields by using Image J software. Non-parametric Wilcoxon Mann-Whiney U-test was applied to assess the statistical significance. Data were presented as mean ± SD. $^{**}P < 0.01$, $^{***}P < 0.001$. N.S. = not significant. (***C***) GFP-LC3 puncta were counted in randomly selected fields by using Image J software. Non-parametric Wilcoxon Mann-Whiney U-test was applied to assess the statistical significance. Data were presented as mean ± SD. $^{*}P < 0.05$, $^{***}P < 0.001$ *vs.* indicated group. N.S. = not significant. (***D***) The mean fluorescence intensity of gB and LC3 in WT Hela cells and Beclin-1 KD Hela cells was quantified in randomly selected fields by using Image J software. Non-parametric Wilcoxon Mann-Whiney U-test was applied to assess the statistical significance. Data were presented as mean ± SD. $^{**}P < 0.01$, $^{***}P < 0.001$, N.S. = not significant.

Figure S6. CORT induces the degradation of PML protein. (***A***, ***B***) Representative immunoblot analysis of endogenous PML. HeLa cells and Hek293 cells were exposed to 50 μM CORT or not for 48 h. Cell lysates were prepared for immunoblot analysis. Immunoblot was performed in two independent experiments. (***C***, ***D***) Immunoblot analysis of exogenously overexpressed PML. HeLa cells and Hek293

cells were transfected with PML-HA for 12 h, then incubated with 50 μM CORT or not for 48 h. Cell lysates were prepared for immunoblot analysis. Immunoblot was performed in two independent experiments, and representative blotting results were shown. (***E*, *F***) Immunoblot analysis of PML, ICP8 and gB. HT22 and TM4 cells were treated with 50 μM CORT or not for 48 h, followed by HSV-1 infection for 24 h. HSV-1 strain F viral titer: 5×10^7 PFU/mL. Expression of the indicated proteins was assessed by immunoblot analysis in two independent experiments.

Figure S7. CORT-driven PML degradation is independent of proteasome pathway. (*A*) Immunoblot analysis of PML. BV2 cells were treated with 10 μM MG132 for 0, 3, 6, 12, and 24 h. Total cellular protein was extracted for immunoblot. (***B***) Immunoblot analysis of PML. BV-2 cells were treated with MG132 (10 μM), CHX (100 μM), or MG132 (10 μM) + CHX (100 μM). Cells were lysed at 12 h and were evaluated via immunoblot analysis. (***C***) The expression of PML was detected. BV2 cells were divided into four groups: Control, CORT (50 μM, 48 h), MG132 (10 μM, 24 h), CORT + MG132 (cells were treated with 50 μM CORT for 24 h before simultaneously incubation with 10 μM MG132 for another 24 h). Total cellular protein was extracted for immunoblot.

Figure S8. CORT-induced autophagy contributes to the degradation of PML. (*A*) Fluorescence staining of endogenous PML. SH-SY5Y cells were transfected with GFP-LC3, and treated with 50 μM CORT or not for 48 h. The white dotted boxes (1 and 2) indicated the areas that were enlarged in the images labeled 1 and 2. The white arrows indicated the co-location of LC3 and PML. Scale bar, 10 μm. (***B***) PML puncta and (***C***) relative fluorescence intensity of PML were quantified. SH-SY5Y cells were exposed to 50 μM CORT or not for 48 h. PML puncta in the nuclei and the relative fluorescence intensity of PML were calculated by using Image J software. Non-parametric Wilcoxon Mann-Whiney U-test was applied to assess the statistical significance. Data were presented as mean ± SD. $^{**}P < 0.01$ *vs.* indicated group. (***D***) The expression of PML was detected. Hela cells were treated with 50 μM CORT for 48 h or not, then cells were harvested in the absence or presence of 50 nM BaF1. Total cellular protein was extracted for immunoblot. (***E***) Immunoblot analysis of PML, gB, ICP27 and LC3. HeLa cells were transiently transfected with scrambled-siRNA (NC) or si-*LC3*, then incubated with 50 μM CORT for 48 h. The two transfected groups were infected with HSV-1 at MOI=1 for another 24 h. HSV-1 strain F viral titer: 5×10^7 PFU/mL. Total cellular protein was extracted for immunoblot.

Figure S9. CORT dampens innate immunity. (*A*) The expression of IFNβ, chemokines, and ISGs were measured by real-time PCR. SH-SY5Y cells were administrated to 50 μM CORT or not for 48 h, followed by HSV-1 infection (MOI=1) for 24 h. One-way ANOVA followed by Tukey's multiple comparisons test

was used to assess the statistical significance. Data were presented as means ± SD of three biological samples. Significances were marked as $^{*}P < 0.05$, $^{**}P < 0.01$, $^{***}P < 0.001$ *vs*. indicated group. (***B***) Immunoblot analysis of IFNβ in mouse hippocampus. Immunoblot was performed in three independent experiments, and representative blotting results were shown. (***C***) Representative immunoblot analysis of cGAS, STING, MAVS, IFNβ, TBK1 and p-TBK1 in SH-SY5Y cells treated with 50 μM CORT for 48 h. Immunoblot was performed in three independent experiments. (***D***) Fold change of the indicated proteins in (***C***) were calculated by Image J software. A non-parametric Wilcoxon Mann-Whiney U-test was used for statistical analyses. Data were presented as mean ± SD of three independent experiments. $^{*}P < 0.05$ *vs.* indicated group. N.S. = not significant.

Figure S10. Representative immunoblot analysis of ICP0. SH-SY5Y cells were subjected to 50 μM CORT or not for 48 h, and followed by HSV-1 infection at MOI=1. HSV-1 strain F viral titer: 5×10^7 PFU/mL. Whole cell lysates were collected at 24 h p.i.. Immunoblot was performed in two independent experiments.

Figure S1.

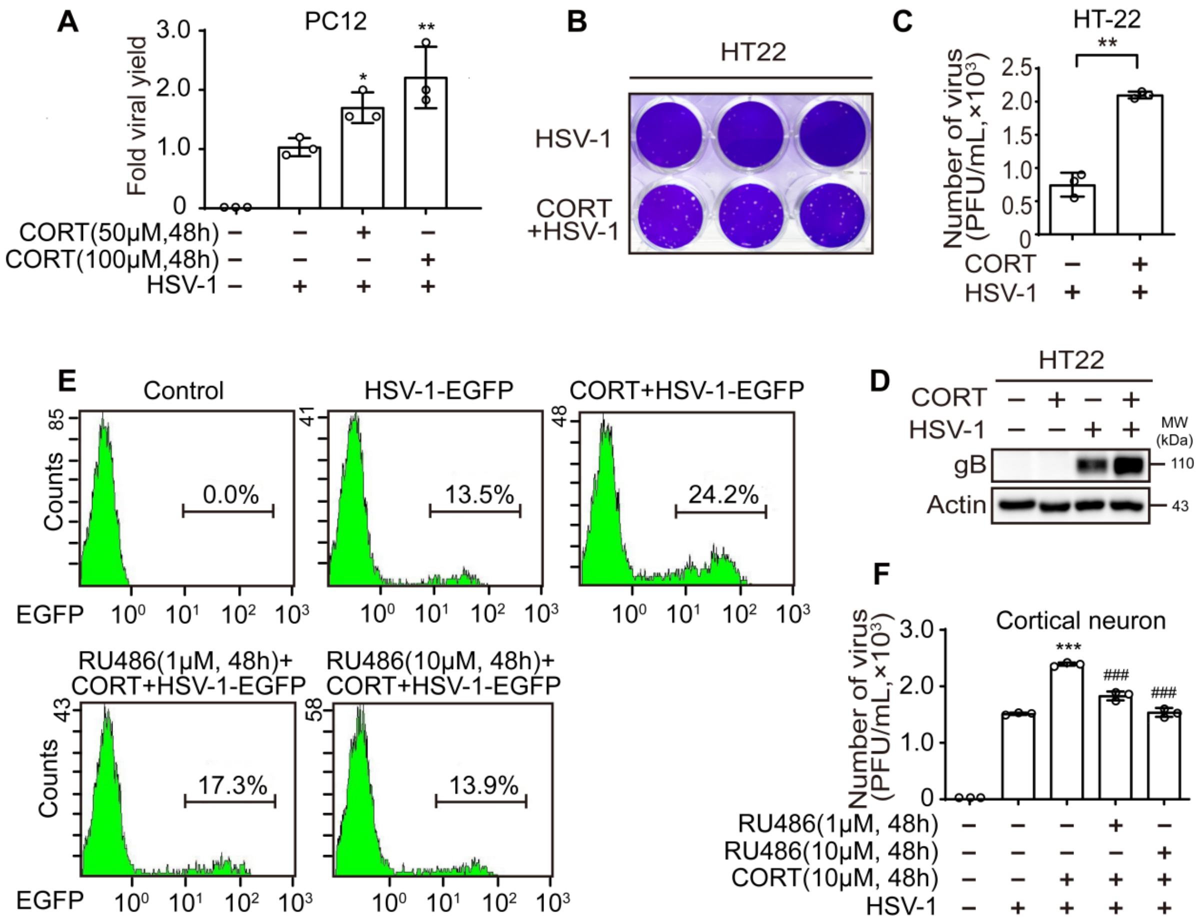
A
PC12
Fold viral yield
CORT(50μM,48h)
CORT(100μM,48h)
HSV-1
B
HT22
HSV-1
CORT +HSV-1
C
HT-22
Number of virus (PFU/mL,×10³)
CORT
HSV-1
D
HT22
CORT
HSV-1
MW (kDa)
gB
Actin
110
43
E
Control
HSV-1-EGFP
CORT+HSV-1-EGFP
Counts
EGFP
0.0%
13.5%
24.2%
RU486(1μM, 48h)+ CORT+HSV-1-EGFP
RU486(10μM, 48h)+ CORT+HSV-1-EGFP
17.3%
13.9%
F
Cortical neuron
Number of virus (PFU/mL,×10³)
RU486(1μM, 48h)
RU486(10μM, 48h)
CORT(10μM, 48h)
HSV-1

Figure S2.

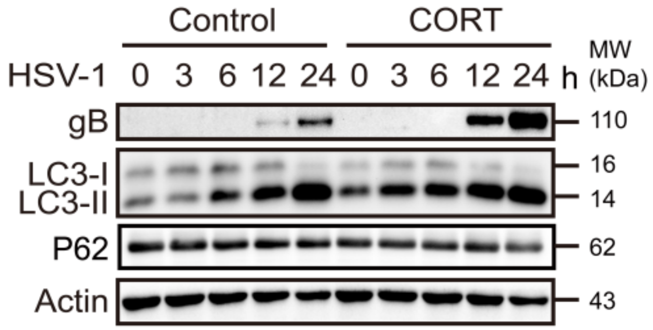
Control
CORT
HSV-1 0 3 6 12 24 0 3 6 12 24 h
MW (kDa)
gB
110
LC3-I
LC3-II
16
14
P62
62
Actin
43

Figure S3.

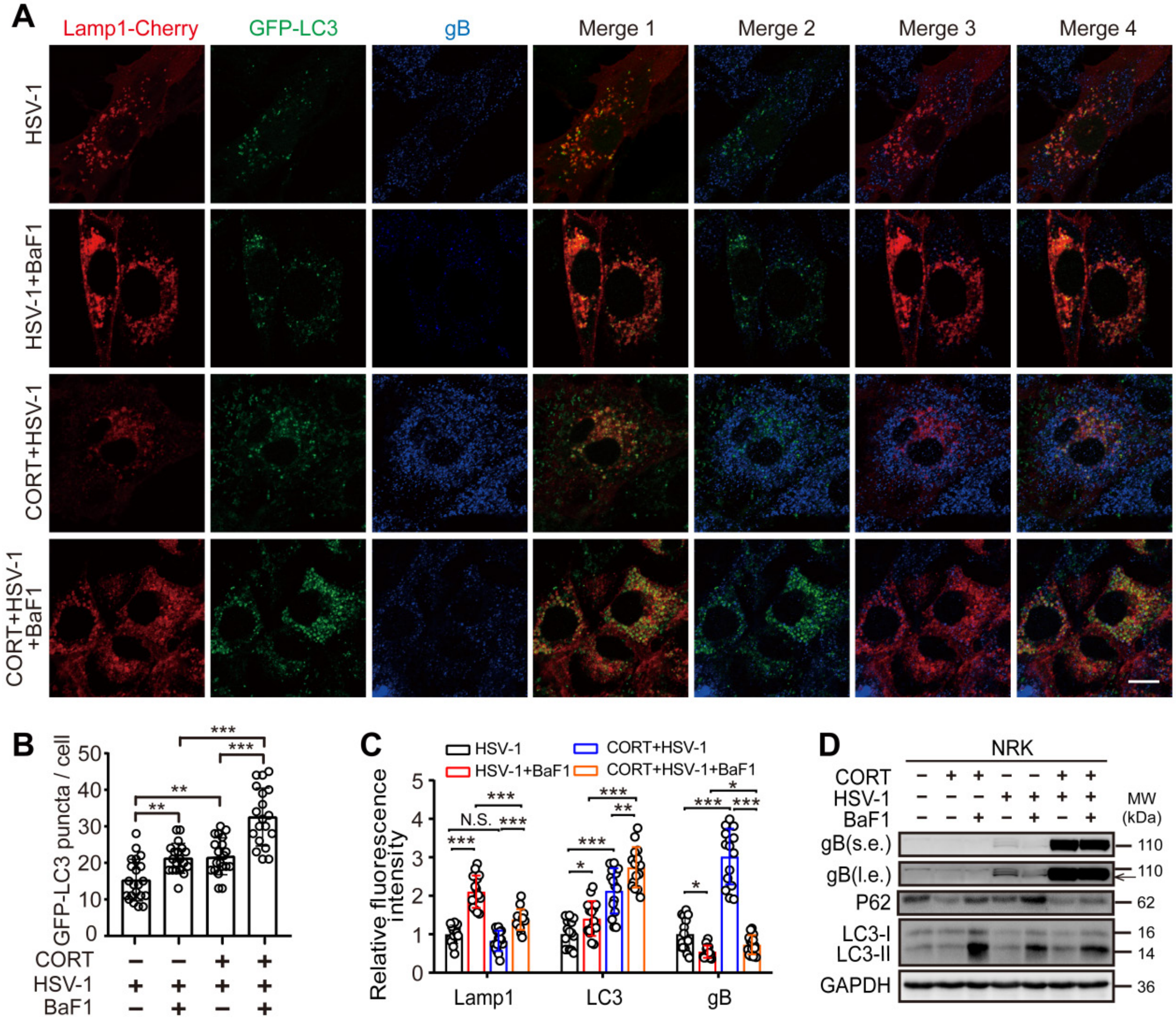
A
Lamp1-Cherry
GFP-LC3
gB
Merge 1
Merge 2
Merge 3
Merge 4
HSV-1
HSV-1+BaF1
CORT+HSV-1
CORT+HSV-1 +BaF1
B
GFP-LC3 puncta / cell
CORT
HSV-1
BaF1
C
HSV-1
HSV-1+BaF1
CORT+HSV-1
CORT+HSV-1+BaF1
Relative fluorescence intensity
N.S.
Lamp1
LC3
gB
D
NRK
CORT
HSV-1
BaF1
MW (kDa)
gB(s.e.)
gB(l.e.)
P62
LC3-I
LC3-II
GAPDH
110
110
62
16
14
36

Figure S4.

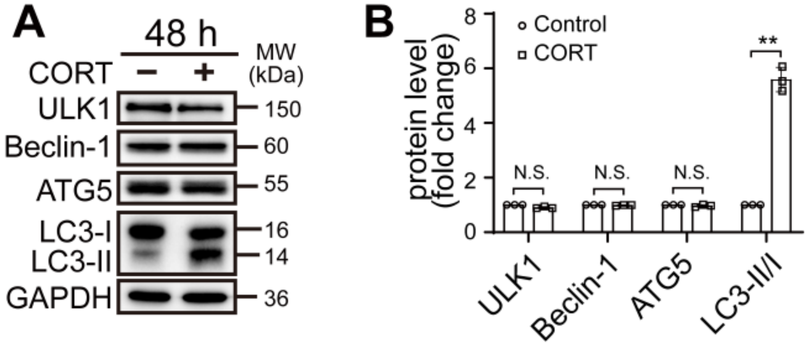

A
48 h
CORT
–
+
MW
(kDa)
ULK1
150
Beclin-1
60
ATG5
55
LC3-I
16
LC3-II
14
GAPDH
36
B
Control
CORT
protein level
(fold change)
8
6
4
2
0
N.S.
N.S.
N.S.
**
ULK1
Beclin-1
ATG5
LC3-II/I

Figure S5.

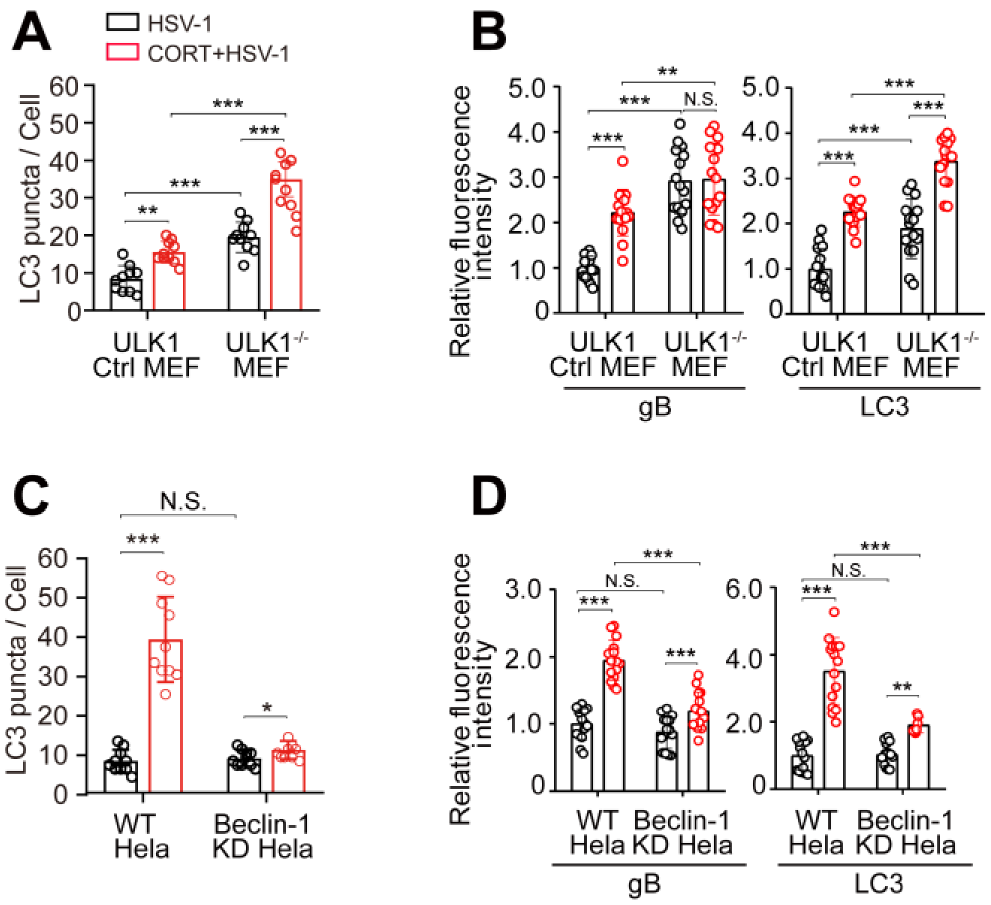

A
HSV-1
CORT+HSV-1
LC3 puncta / Cell
ULK1 Ctrl MEF
ULK1-/- MEF
B
Relative fluorescence intensity
ULK1 Ctrl MEF
ULK1-/- MEF
gB
LC3
N.S.
C
LC3 puncta / Cell
N.S.
WT Hela
Beclin-1 KD Hela
D
Relative fluorescence intensity
N.S.
WT Hela
Beclin-1 KD Hela
gB
LC3

Figure S6.

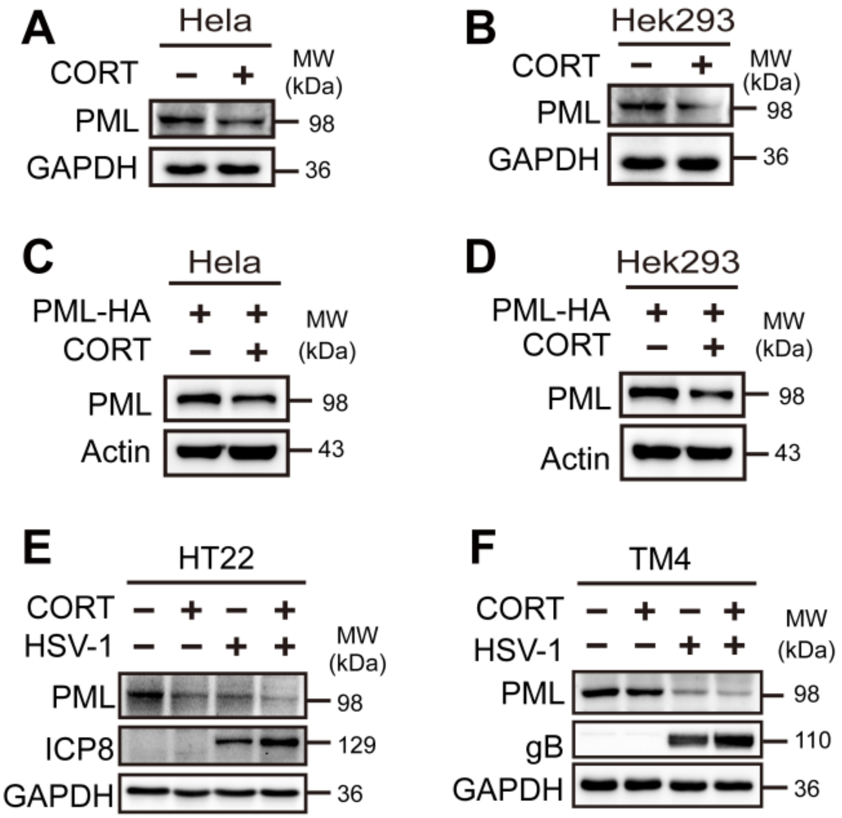
A
Hela
CORT
MW (kDa)
PML
98
GAPDH
36
B
Hek293
CORT
MW (kDa)
PML
98
GAPDH
36
C
Hela
PML-HA
CORT
MW (kDa)
PML
98
Actin
43
D
Hek293
PML-HA
CORT
MW (kDa)
PML
98
Actin
43
E
HT22
CORT
HSV-1
MW (kDa)
PML
98
ICP8
129
GAPDH
36
F
TM4
CORT
HSV-1
MW (kDa)
PML
98
gB
110
GAPDH
36

Figure S7.

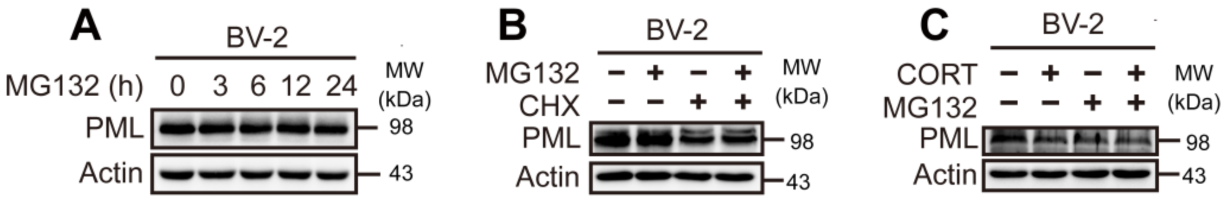

A
BV-2
MG132 (h) 0 3 6 12 24
MW (kDa)
PML 98
Actin 43
B
BV-2
MG132 − + − +
CHX − − + +
MW (kDa)
PML 98
Actin 43
C
BV-2
CORT − + − +
MG132 − − + +
MW (kDa)
PML 98
Actin 43

Figure S8.

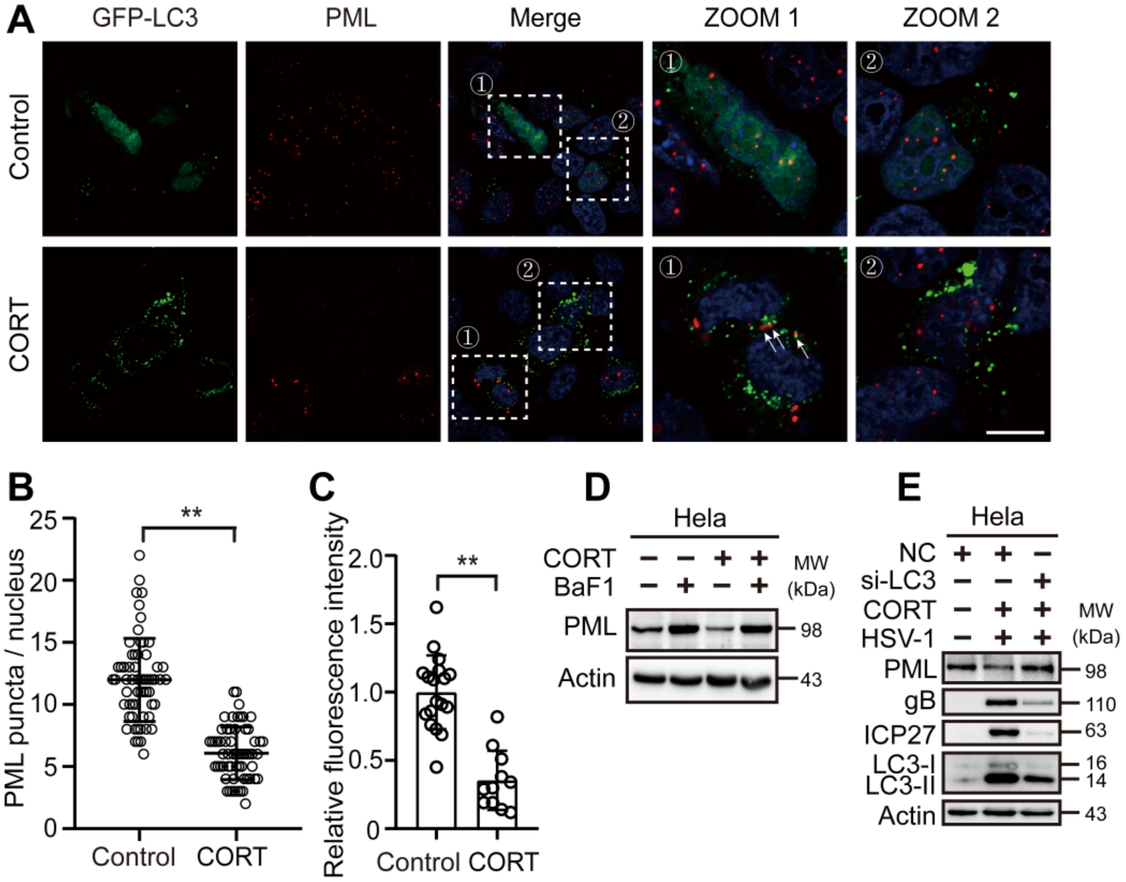
A
GFP-LC3
PML
Merge
ZOOM 1
ZOOM 2
Control
CORT
B
PML puncta / nucleus
25
20
15
10
5
0
**
Control
CORT
C
Relative fluorescence intensity
2.0
1.5
1.0
0.5
0
**
Control
CORT
D
Hela
CORT
BaF1
MW
(kDa)
PML
98
Actin
43
E
Hela
NC
si-LC3
CORT
HSV-1
MW
(kDa)
PML
98
gB
110
ICP27
63
LC3-I
LC3-II
16
14
Actin
43

Figure S9.

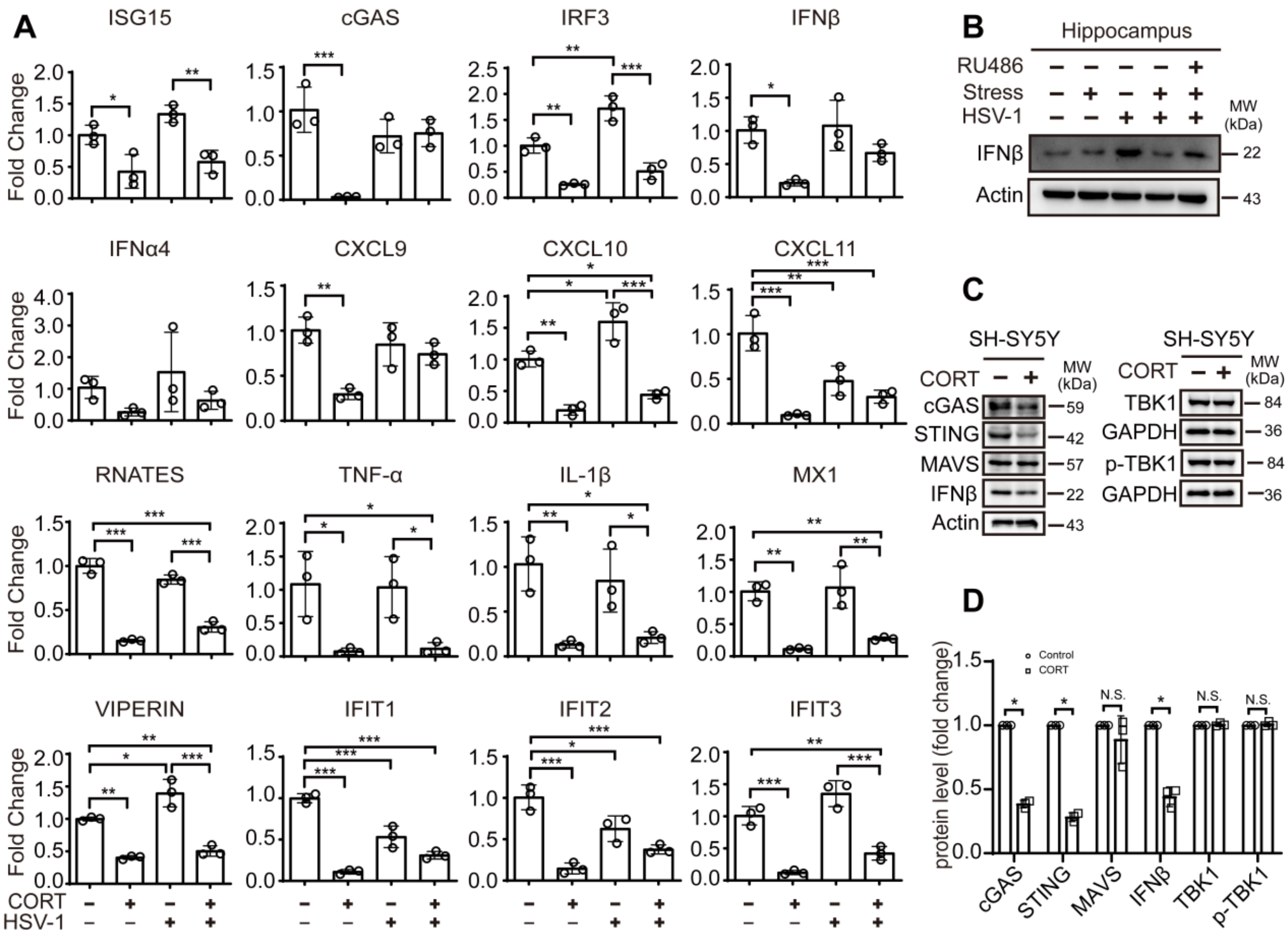
A
ISG15
cGAS
IRF3
IFNβ
IFNα4
CXCL9
CXCL10
CXCL11
RNATES
TNF-α
IL-1β
MX1
VIPERIN
IFIT1
IFIT2
IFIT3
Fold Change
CORT
HSV-1
B
Hippocampus
RU486
Stress
HSV-1
MW (kDa)
IFNβ
Actin
C
SH-SY5Y
CORT
cGAS
STING
MAVS
IFNβ
Actin
TBK1
GAPDH
p-TBK1
D
Control
CORT
protein level (fold change)
N.S.
cGAS
STING
MAVS
IFNβ
TBK1
p-TBK1

Figure S10.

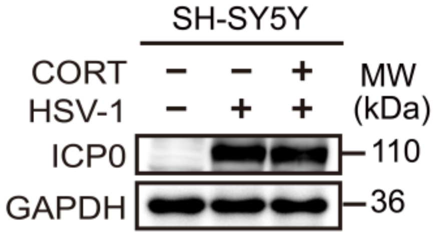

Supplement: Supplementary file 1 — Supplementary figures. [file thnov10p9032s1.pdf]
